# Long-read trio sequencing of individuals with unsolved intellectual disability

**DOI:** 10.1038/s41431-020-00770-0

**Published:** 2020-11-30

**Authors:** Marc Pauper, Erdi Kucuk, Aaron M. Wenger, Shreyasee Chakraborty, Primo Baybayan, Michael Kwint, Bart van der Sanden, Marcel R. Nelen, Ronny Derks, Han G. Brunner, Alexander Hoischen, Lisenka E. L. M. Vissers, Christian Gilissen

**Affiliations:** 1 Department of Human Genetics, Radboud University Medical Center, Nijmegen, The Netherlands; 2 Radboud Institute for Molecular Life Sciences, Radboud University, Nijmegen, The Netherlands; 3 Pacific Biosciences, Menlo Park, CA USA; 4 Donders Institute for Brain, Cognition and Behaviour, Radboud University, 6525 HR Nijmegen, The Netherlands; 5 Department of Clinical Genetics, Maastricht University Medical Center, Maastricht, The Netherlands; 6 Department of Internal Medicine, Center for Infectious Diseases (RCI), Radboud University Medical Center, Nijmegen, The Netherlands

**Keywords:** Structural variation, DNA sequencing

## Abstract

Long-read sequencing (LRS) has the potential to comprehensively identify all medically relevant genome variation, including variation commonly missed by short-read sequencing (SRS) approaches. To determine this potential, we performed LRS around 15×–40× genome coverage using the Pacific Biosciences Sequel I System for five trios. The respective probands were diagnosed with intellectual disability (ID) whose etiology remained unresolved after SRS exomes and genomes. Systematic assessment of LRS coverage showed that ~35 Mb of the human reference genome was only accessible by LRS and not SRS. Genome-wide structural variant (SV) calling yielded on average 28,292 SV calls per individual, totaling 12.9 Mb of sequence. Trio-based analyses which allowed to study segregation, showed concordance for up to 95% of these SV calls across the genome, and 80% of the LRS SV calls were not identified by SRS. De novo mutation analysis did not identify any de novo SVs, confirming that these are rare events. Because of high sequence coverage, we were also able to call single nucleotide substitutions. On average, we identified 3 million substitutions per genome, with a Mendelian inheritance concordance of up to 97%. Of these, ~100,000 were located in the ~35 Mb of the genome that was only captured by LRS. Moreover, these variants affected the coding sequence of 64 genes, including 32 known Mendelian disease genes. Our data show the potential added value of LRS compared to SRS for identifying medically relevant genome variation.

## Background

In the last decade short-read sequencing (SRS) approaches, such as whole exome sequencing (WES) and more recently whole genome sequencing (WGS), have revolutionized the field of medical genetics. Especially for clinically and genetically heterogenic disorders, such as intellectual disability (ID), WES has become the method of choice, allowing the identification of the underlying genetic defect in 40–60% of patients [1]. A substantial fraction (25–30%) of the diagnostic success is due to recent progress in the discovery of new genes underlying disease [2–4].

It has however been shown that, due to technical limitations, SRS approaches often lack sensitivity and specificity for a large proportion of structural variants (SVs) [5–7]. These limitations can be overcome by long-read sequencing (LRS). For instance, recent LRS studies have revealed that each human genome harbors thousands of SVs, in total spanning more than 10 Mb, that have largely remained undetected with conventional SRS [6, 8–11]. In addition, LRS of a haploid human genome resulted in SV call-sets three to sevenfold larger than those produced by standard SRS studies such as the 1000 genomes project [12]. This makes SVs an even more important source of human genome variation than anticipated so far, accounting for the greatest number of divergent bases across the human genome [13, 14].

SVs, in particular copy number variations (CNVs), have long been recognized as an important cause for severe human diseases [15–17]. As inversions and translocations were more difficult to study in a genome-wide fashion with good resolution, their biological and clinical relevance is likely underestimated so far [18].

There have now been some examples of studies in which LRS was applied to individual patients to resolve the genetic origin of disease [19, 20] (see also ref. [21] for an overview). In addition to higher yield of SVs, these studies indicate that LRS allows researchers to study genomic regions that are often challenging to sequence with SRS [22]. Therefore, we hypothesized that LRS may also enhance clinical diagnosis in an unbiased and genome-wide fashion for patients whose genetic etiology remained elusive after SRS WES and WGS approaches. To test this hypothesis, we here use a trio-based LRS approach for five individuals with unresolved ID and their parents [23], and compare LRS and SRS results to determine the added value of LRS for identifying all medically relevant genome variation in a single experiment.

## Materials and methods

### Patient inclusion

Five patients with severe ID and their parents were selected for this study. All five patients were born to non-consanguineous parents with a negative family history. All were diagnosed with severe developmental delay, and co-morbidities, including epilepsy and/or behavior problems. In addition, three of them showed facial dysmorphisms frequently observed in patients with genetic disorders (Supplementary Clinical Notes). Prior testing to detect the genetic cause of disease included genomic microarray [24], exome sequencing [25], genome sequencing [23], and methylome analysis [26], which had not resulted in a molecular diagnosis (Table S1). This study was conducted and approved by the Institutional Review Board of the Radboud university medical center (2017–3831).

### Genomic DNA extraction, shearing, and library preparation for LRS

Genomic DNA was extracted from whole blood using the Qiagen (Hilden, Germany) Puregene Blood Core Kit C. gDNA integrity was assessed with pulsed-field gel electrophoresis 115 ng/well, 17 h runtime at 70 V (Fig. S1). gDNA was sheared with the Diagenode (Liege, Belgium) Megaruptor using long hydropores. A total of 12 µg gDNA was sheared to 60 Kb fragments in a total volume of 300 µl using the preinstalled settings. DNA was concentrated using 0.45× bead/sample ratio of Ampure PB beads and was eluted in 73 µl elution buffer. Qubit dsDNA BR assay was used to quantify DNA concentration.

All libraries were prepared using SMRTbell™ Template Prep Kit 1.0, according to the Procedure & Checklist—Preparing >30 Kb SMRTbells™ (Pacific Biosciences, Menlo Parc, CA, USA). As 10 µg DNA input was used instead of 5 µg, all reaction volumes were doubled until the size-selection step. DNA was sheared using the Megaruptor^®^, after which size selection was performed using the BluePippin high-pass DNA size selection with 0.75% DF marker U1 high-pass 30–40 Kb v3 cassette. The range selection mode was set from 25 to 80 Kb. After size selection, Ampure PB bead cleanup steps were performed using 1× bead/sample ratio. DNA damage repair after size selection was performed with the reaction volumes described in the protocol. Qubit dsDNA HS assay was used to quantify DNA concentration.

### Long-read sequencing

Sequencing primer v3 was annealed to the SMRTbell™ library. Polymerase was bound using the Sequel Binding Kit 2.0. SMRTbell™ complexes were purified using the Procedure & Checklist—Sample Clean-up using MicroSpin™ columns S-400 for diffusion loading. Sequencing reaction was performed using the Sequel sequencing plate 2.0 on a SMRT-cell 1M chip. On plate sample concentration was 10 pM, movie time was set to 600 min with an immobilization time of 120 min.

For four trios (Trio 1, 2, 3, 4) sequencing was performed at Radboud university medical center using a Pacific Biosciences (Pacbio) Sequel I System. In total 145 SMRT Cells 1M were used, resulting in an average genome coverage of ~15× (Table S2). A single trio (Trio 5) was sequenced using a Sequel I System at Pacific Biosciences to an average genome coverage of 40× using 89 SMRT Cells 1M (Table S2).

For analyses on the percentage of the genome with specific fold-coverage we split the genome into “easily accessible” and “difficult” regions, and made the percentage calculations disregarding the latter. As “difficult” regions we defined those annotated as “scaffold”, “contig”, “clone”, “telomere”, “centromere”, or “heterochromatin” in the GRCh38 assembly. Additionally, chrY was included in the “difficult” regions to enable better comparison between samples of different gender.

### Variant calling from LRS data

For each trio, long reads were aligned to the GRCh38 reference genome (version GCA_000001405.15_GRCh38_no_alt_plus_hs38d1_analysis_set, with all non-primary contigs concatenated), using minimap2 (2.11-r797) with parameters “-a --eqx -L -O 5,56 -E 4,1 -B 5 –secondary = no -z 400,50 -r 2k -Y” [27]. SVs were called using PBSV (https://github.com/PacificBiosciences/pbsv) (version 2.1.0) with default parameters for both the “discover” and “call” steps of PBSV’s workflow. The “call” step was run jointly on all 15 samples. PBSV is most effective for insertions with sizes from 50 bp to 5 Kb, deletions with sizes from 50 bp to 100 Kb and inversions with sizes from 200 bp to 5 Kb. Therefore, here we only considered variants of 50 bp and above as SVs. We annotated our SV calls using AnnotSV (version 2.0) [28].

Single nucleotide substitution variants (SNVs) were identified with Longshot (0.2.0) [29] using default parameters except for parameter max_cov, which was set to 50 for Trios 1, 2, 3, and 4 and 100 for Trio 5, in order to improve analysis runtime. The output was annotated with Annovar (2018-04-16) [30], using the core annotate_variation.pl script with RefGene hg38 build. We then compared VCF files for identifying SNVs that are uniquely detected by LRS or SRS using a custom script.

Read and mapping metrics were obtained from the aligned BAM files using SAMtools, Qualimap [31] and manual processing (Table S2). The number of sequenced bases was obtained from the “total length” field of the SAMtools’ stats subcommand output. From the same output, the field “bases mapped” was divided by “total length” and by 3 × 10^9^ in order to estimate the percentage of bases mapped and the mean coverage, respectively. The mean, median, and N50 of read lengths were calculated by filtering out non-primary alignment, duplicate reads and supplementary alignments from the BAM files, and then manually processing the remaining mapped reads using awk and R. The mean mapping quality and error rate were calculated using Qualimap.

### SRS and variant calling

Whereas all five trios were previously exome and genome sequenced using SRS technology (Table S1) [23, 32], Trio 5 was re-sequenced using 2 × 150 bp paired-end reads on the Illumina NovaSeq 6000 instrument to an average coverage of 29× to allow for comparison of LRS to today’s standard whole genome SRS. Reads were aligned using BWA mem (version 0.7.12-r1039) and SNVs were called using the xAtlas caller with default parameters (version 0.1) [33], and only variants passing all filters were retained. SVs were identified using three different variant callers: Manta (version 1.1.0) [34], LUMPY (version 0.2.13) [35], and DELLY (version 0.7.8) [36]. The output of LUMPY and DELLY were genotyped using SVTyper (version 0.6.0) [37]. SVs smaller than 50 bp in size were filtered out, and minimum genotype quality was set to 20.

### Quality assessment of SVs and SNVs

For each trio, we quantified the Mendelian inheritance errors (MIEs) in SV calls in the LRS data. For this analysis we used only deletions, insertions, and inversions. MIEs were quantified using the plugin “mendelian” from BCFtools (version 1.9). For comparison, we performed the same analysis on SVs detected with SRS in Trio 5.

Furthermore, for each proband, we identified SVs from LRS that were uniquely found in that proband and not in any of the other probands. For these SVs, we looked for MIE using BCFtools (version 1.9) plugin “mendelian”. Similarly, we performed a Mendelian concordance analysis of SNVs with vcftools –mendel option. Only filtered SNVs on autosomal chromosomes were considered for this analysis. Specifically, for Trio 5, we analyzed both Longshot calls from LRS as well as xAtlas calls from SRS.

### Comparison of sequence coverage between short and long-read sequencing

In Trio 5, we identified genomic regions that had no coverage with SRS but were well-covered by LRS using BEDtools genomecov (version 2.25.0) [38]. For this, we considered only reads with a minimum mapping quality of 10 and used Gencode Basic gene set, release 21 to define genic and exonic regions. We determined what percentage of these regions without coverage are in telomeric and centromeric sequences. We obtained a set of telomeric and centromeric regions from UCSC genome browser and used BEDtools intersect (2.25.0) to find overlaps.

We also determined genes that were not well-covered by SRS using these Gencode annotations. A gene was considered not well-covered when at least 10% of its length was not covered by SRS. We then compared the GC-content of these genes to a randomized set of well-covered genes with comparable lengths. GC-percentages of all genes were obtained from the UCSC Genome Browser.

### Comparison of SVs

The SV call sets from Manta, LUMPY and DELLY from SRS data were each compared to the LRS SV calls of Trio 5. The comparisons were run on large (≥50 bp) deletions, insertions, and inversion, using Truvari (version 1.3.2). Truvari matches SVs between different datasets. For a match, the maximum distance between start and end coordinates of two SVs was set to 1 Kb with parameter –refdist. Additionally, by default, two calls should be of the same type and the ratio of the size of the smaller call over the larger call should be at least 0.7 for a match. The parameter --pctsim was set to 0 as it can only be applied on sequence-resolved variants. Parameter --sizemax was set to 100 Mb to circumvent the default 50 Kb.

The SV call sets of Manta, LUMPY and DELLY were also compared to each other to examine the concordance between the three methods. The comparison was done with Truvari (version 1.3.2) for all SV types and sizes, using similar parameters as described above.

We also compared our SV call set with two published datasets that were also produced by PacBio instruments:

- We used SV calls available from Pacific Biosciences for the HG002 reference sample from the Genome in a Bottle consortium, sequenced on a PacBio Sequel instrument at 10× coverage (https://downloads.pacbcloud.com/public/dataset/HG002/Sequel-201810/). The comparison was done using Truvari for deletions, insertions, and inversions larger than 50 bp and passing all filters [39].
- In addition, we compared our LRS SV dataset to the results obtained by Audano et al. [40] based on long-read WGS sequencing of 15 individuals across different populations. As Audano et al. only supplied SV calls in bed format, the comparison for overlap was performed using BEDtools (2.25.0) using a 50% reciprocal overlap setting (https://ars.els-cdn.com/content/image/1-s2.0-S0092867418316337-mmc1.xlsx).

### Identification of de novo SVs

For each trio, potential de novo SVs were identified based on the genotyped SV calls of PBSV. Initially, we selected all variants with a heterozygous genotype (e.g., one wild-type allele and one allele with SV) in a proband, and a homozygous reference genotype (e.g., two wild-type alleles) in both parents. These variants were then subjected to additional filtering in order to identify high-quality de novo candidates:

- Heterozygous alternative allele only in proband.
- Read ratio supporting alternative allele between 0.3 and 0.7 in proband.
- Minimum depth of coverage of six reads at SV coordinates for all samples.
- Homozygous for the reference allele in all other samples.
- Zero reads supporting alternative allele in other samples.

Each candidate de novo SV was visually inspected using the Integrated Genomics Viewer (IGV, version 2.4.14) [41], which adds better support for visualizing LRS, such as grouping and coloring of alignments based on ZMW or sequencing movie name, and allows for better identification of false positive SV calls due to sequencing artifacts. The subset of variants passing visual inspection was sent for PCR validation.

### Identification of de novo SNVs

For each trio, potential de novo SNVs were identified from LRS data using VCFtools (0.1.13) –mendel, which produces a list of MIEs. We selected a quality score cutoff of 30 for missense variants such that all previously identified de novo mutations were included in the result. Filtered missense variants and all loss-of-function variants were visually inspected in IGV before being sent for validation with Sanger sequencing.

### Recessive inheritance analysis

We used a custom script to parse AnnotSV annotations and identify genes that are affected by homozygous or compound heterozygous SVs and SNVs affecting the coding regions in all trios. Only loss-of-function SNVs with quality score higher than 30 were considered for this analysis.

### Validation of SV and SNV events in LRS

Candidate de novo SVs and SNVs were visually inspected in the BAM files of the patient as well as both parents by using the Integrative Genomics Viewer (IGV). Based on the examination of the mode of inheritance, read quality, and mapping quality, each variant was classified as follows:

1. Inherited variant: even though the variant was classified as possible de novo event, the BAM files showed that the variant was also present in one of the parents (e.g., missing call in the parental data).
2. False positive variant: the quality and mapping of the reads at the region of the variant was substandard.
3. The variant appears as a true de novo event.

The remaining candidate de novo SVs were subsequently validated by breakpoint spanning PCRs and evaluation by Agarose Gel Electrophoresis. Primers were designed using Primer3. PCRs were performed by using Amplitaq Gold 360 Master Mix (Thermo Fisher Scientific) or Phusion Hot Start (Finnzymes) both according to the manufacturer protocol. The agarose gels were visually inspected to assess whether the SVs appeared to be genuine de novo events. Hereto, it firstly needed to show a second PCR product representing the variant allele, next to the product of the expected size for the wild-type allele, and secondly, the second PCR product was only to be present in the proband and absent in the respective parents, indicating a de novo event.

The SNVs retained as potential de novo events were validated using Sanger sequencing. Primers were designed using Primer3Input. PCRs were using Amplitaq Gold 360 Master Mix (Thermo Fisher Scientific) according to the manufacturer protocol. PCR products were enzymatically cleaned by using Exonuclease I and FastAP, after which samples were Sanger sequenced. Finally, Sanger sequencing traces were analyzed using the VectorNTI software package (Thermo Fisher Scientific).

### Titration analysis of LRS

Titration analysis was performed by subsampling in silico the LRS data of samples from Trio 5. Subsampling was done on the BAM files using SAMtools view (version 1.6) and the –s parameter to pass the desired fraction of data to retain. The original LRS data were subsampled to coverages 30×, 20×, 15×, 10×, and 5×. Each subsampled BAM file was then used for SV discovery with PBSV (version 2.1), and SV calling was performed jointly for the trio at each coverage.

The SV call set of Trio 5 proband at each coverage was compared to the SV calls of the original full coverage dataset. The comparison was performed using Truvari (version 0.4) with parameters: --multimatch, --passonly, --pctsim 0, and --refdist 1000. Parameters --sizemin and --sizemax were set to 50 and 1,000,000.

We performed a similar analysis for SNV calling and used the same subsampled BAM files as described above, and ran Longshot to call SNVs at different coverages. We then used the original full coverage SNV calls as a truth set to calculate precision and recall values for each level of coverage.

## Results

### Long-read WGS characteristics

Five patient-parent trios were subjected to LR-WGS. From all sequenced reads per sample, the read mapping rate varied between 94.5 and 98.7% (Table S2). Whereas the longest read obtained was 60 Kb in size, the average read length was 9.5 Kb (N50 average: 17481.5 Kb; Fig. S2). The error rate was consistent across samples (0.16 errors per aligned base), which is in line with what is reported in the literature [42]. Sequencing and mapping resulted in an average coverage of 16.01× for Trios 1, 2, 3, and 4, and 39.77× for Trio 5 (Table 1). On average 99.9% of the easily accessible genome regions were covered at least 5× and more than 85.4% of the complete genome including decoy sequences, centromeres, assembly gaps and chromosomes Y and M (Tables S2 and S3).

**Table 1:** Overview of samples, sequencing statistics, identified variation, and Mendelian inheritance errors.

Sample	Coverage (×)	# SVs	Total affected sequence (bp)	SV MIE (%)	Unique SVs in cohort	Study-specific SVs	SNV	SNV MIE (%)
T1P	15.7	29,030	12,775,459	4409	75	18,601	3,142,916	448,808
T1F	12.6	26,475	11,340,949	(15.2%)	245	14,172	2,663,675	(14.3%)
T1M	14.9	27,421	12,998,704		236	18,334	3,007,973	
T2P	17.3	28,766	12,974,306	3186	81	19,098	3,383,890	290,676
T2F	14.2	27,412	12,595,695	(11.1%)	244	19,549	3,127,690	(8.6%)
T2M	17.2	28,649	12,851,920		253	18,539	3,353,748	
T3P	15.0	28,111	12,689,520	2681	57	19,321	3,147,660	219,392
T3F	18.3	28,971	13,327,881	(9.5%)	247	19,198	3,382,461	(7.0%)
T3M	18.0	28,962	12,884,749		264	19,419	3,352,767	
T4P	16.1	28,640	12,830,123	2763	33	19,470	3,166,126	301,415
T4F	16.7	28,746	12,540,739	(9.6%)	261	21,653	3,261,812	(9.5%)
T4M	16.1	28,322	12,683,375		256	19,523	3,101,729	
T5P	41.6	33,056	14,085,392	2130	16	21,539	3,956,435	125,023
T5F	37.6	33,277	14,235,204	(6.4%)	228	18,974	3,905,927	(3.2%)
T5M	40.1	33,138	13,949,579		273	22,023	3,932,800	

Columns from (left to right) indicate: sample identifier, average coverage across the genome (GRCh38), number of identified SVs (≥50 bp), total number of based affected by SVs, number of SVs in proband with a Mendelian inheritance error (% is indicated below), number of SVs only occurring in this sample, number of SVs only found in this study (not in HG0002 and Audano et al.), number of identified SNVs, number of SNVs in proband with a Mendelian inheritance error (% is indicated below).

*F* father, *M* mother, *P* proband, *SV* structural variant, *MIE* Mendelian Inheritance Error, *SNV* single nucleotide variant.

### Structural variation across the cohort

We identified an average of 28,292 SVs (≥50 bp) per sample for Trios 1, 2, 3, 4. For Trio 5, sequenced at higher depth, we identified 33,157 SVs (Tables 1, S4a, Fig. S3), suggesting that greater sequencing depth enhances sensitivity for SV detection. Across all 15 samples, we identified 55,025 unique SVs, including 34,690 insertions (63%), 20,307 deletions (37%), and 28 inversions (0.05%) (Table S4b). There was a gradual decline of SVs abundance with increasing size, with smaller SVs being more abundant than larger SVs. Exceptions were noted for SVs at ~300 bp, representing *Alu* short interspersed nuclear elements and those at ~6.4 Kb representing LINE1 long interspersed nuclear elements (LINEs; Fig. S4a, b).

All detected SVs affected in total ~13 Mb of genome sequence per sample (deletions: 6.5 Mb, insertions: 6.4 Mb, and inversions: 35.8 Kb, Table S5). On average, 173 SVs per individual affected the coding sequences, including a total of 81 genes with a known disease relationship according to OMIM (Table S6). However, none of these SV events could be linked to the patient phenotype. For Trio 5, we performed subsampling of the LRS data to determine the effect of coverage on SV discovery. As expected, we found that SV yield increases with coverage, but that the number of additional SVs diminishes beyond 10×.

### Overlap with published datasets

We compared our results to other published LRS datasets based on the GRCh38 reference genome, and sequenced using Pacific Biosciences Sequel instrument. First, we used SV calls from the HG002 reference sample that was sequenced at tenfold depth [39]. We found that from the HG002 dataset 77.4% (*n* = 5,920) of the deletions and 73.9% of the insertions (*n* = 7,043) were also detected in our dataset, showing a high degree of overlap (Table S7). We also compared our SV calls with the published study of Audano et al. [40], whose work included the genomes (50× coverage) from 13 diploid individuals, the majority of whom were of non-European descent. Almost 80% of the variants they reported were not previously published to that date. We found that 33% of deletions (*n* = 4,526) and 73% of insertions (*n* = 15,963) in our cohort were novel compared to Audano et al. (Table S8), highlighting the fact that there is still a large degree of previously hidden structural variation to be identified in the human genome.

### Comparison of SRS and LRS structural variation

**Fig. 1:**
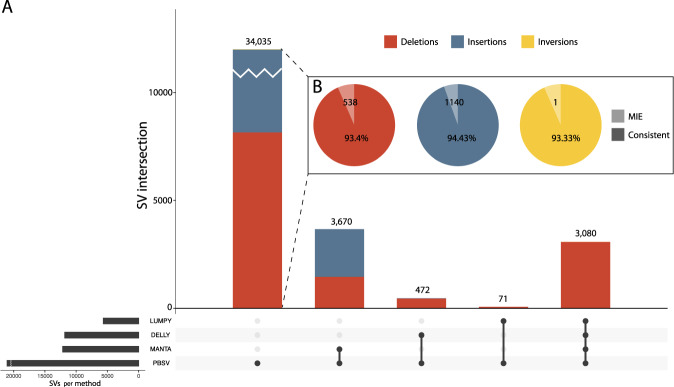
Comparison of structural variants called with long-read sequencing and short-read sequencing. **A** comparison of structural variants identified in Trio 5 between long-read sequencing using PBSV and short-read sequencing using three algorithms for structural variant detection. The plot depicts the number of different structural variants that were identified by each combination of methods, indicated below the corresponding bar. Deletions in red, insertions in blue, and inversions in yellow. The bottom left bar plot depicts the total number of SVs identified with each method. **B** Pie charts show the number of mendelian inheritance errors for the three types of SVs identified by LRS and the percentage of concordant calls.

In order to compare the performance of LRS and SRS, Trio 5 was sequenced on an Illumina NovaSeq 6000 instrument to an average coverage of 29× (Tables S9 and S10). SV calls for SRS were obtained by three different calling algorithms: Manta, LUMPY, and DELLY. We compared each of the SRS SV call sets separately to the SVs from LRS, considering only deletions, insertions, and inversions (Fig. 1). Between 51 and 78% of deletions identified in SRS were detected in LRS whereas only 25–38 % of deletions in LRS were detected in SRS by any of the three different calling algorithms (Table S11). For insertions, 83–91% of calls detected in SRS were detected in LRS but only 1–9% of insertions from LRS were also detected in SRS. The large differences in concordance between the results of the three different SRS SV calling algorithms and SVs from LRS emphasizes the challenges of SV detection based on SRS.

### Quality of SV calls based on Mendelian inheritance

**Fig. 2:**
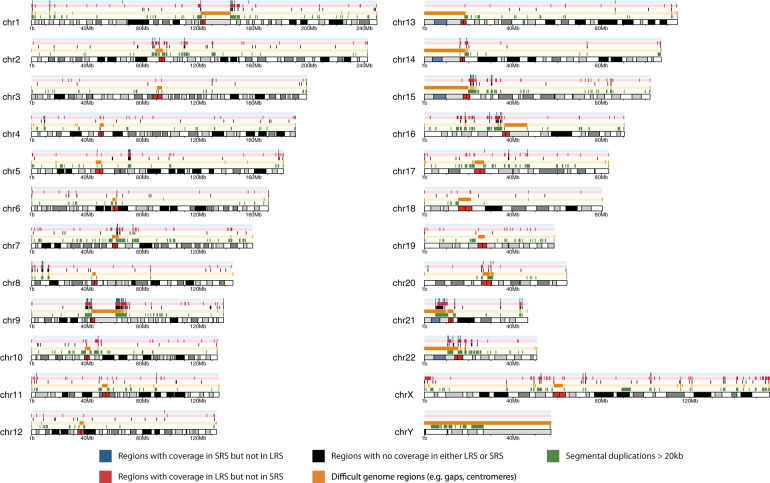
Schematic digital ideogram depicting genomic regions larger than 1 Kb without LRS or SRS coverage. From top to bottom tracks indicate: regions with sequence coverage in SRS but no coverage in LRS (blue); regions with sequence coverage in LRS but no coverage in SRS (red); regions with no sequence coverage in neither LRS and SRS (black); genome regions that are difficult to assess like centromeres, telomeres, and gaps (orange); regions with segmental duplications larger than 20 Kb (green). Note that blue, red, black, and orange regions are mutually exclusive but that suggestive overlap from the figure is due to the limited resolution.

Our trio-based sequencing design allowed us to assess the Mendelian inheritance of SV calls. We define MIEs as SNVs in a proband that could not have been inherited from either parent, resulting in a genotype that is inconsistent with Mendelian transmission. MIEs are commonly attributed to erroneous sequencing calls [43]. Conversely, proper Mendelian inheritance of SVs lends additional support to their reliability. We found that more than 90% of SV calls were concordant with Mendelian inheritance within Trios 2, 3, and 4 (Fig. 2, Table S12). We obtained a lower concordance (87%) for Trio 1, likely due to a lower coverage in the father (12.6×) and, consistent with this, highest concordance in Trio 5 (96%) that was sequenced at higher coverage (39×). Moreover, in comparison to SRS of the same samples (Trio 5), LRS had a comparable overall percentage of MIEs to SRS (~5%). If we consider Mendelian concordant SVs detected by either technology as our truth set, then LRS has almost five times higher sensitivity than SRS (93% vs. 19%, respectively).

The high quality of the SV calls is also apparent from the unique SV events that were only identified within a single trio in the proband, but in none of the other trios. Mendelian inheritance concordance for unique deletions was 90.7%, similar to the overall concordance (Table S13). However, for unique insertion events concordance was only 76.0% suggesting that detection of these events is more challenging in LRS data.

### De novo SV discovery

De novo mutations are a well-known cause of ID [1]. We therefore set out to filter our dataset for de novo SVs based on SV calling genotypes and minimal quality criteria. On average, this led to the identification of ten candidate de novo SVs per trio (range 2–17; Table S14). After visual inspection of read mappings, the number of candidates was manually curated to a set of eight possible de novo SVs ranging between 0 and 3 de novo candidates per trio (Table S15, Fig. S5). Notably, the candidate de novo SVs that were removed after inspection were mostly false positive SV calls due to repetitive sequence, or inherited as a consequence of a missed SV call in one of the parents. In accordance with the hypothesis that increased sequencing coverage results in increased specificity, the lowest number of de novo candidates was found in the high coverage Trio 5.

### De novo SV validation

Systematic validation of the eight potential de novo SVs left in trios 1–4 after visual inspection and follow-up by breakpoint spanning PCRs, four SVs were confirmed as genuine SV events, for two others the PCRs remained inconclusive (non-unique or no PCR product), and two SVs were a likely false positive call from LRS SV data. None of the genuine SV events were however of de novo origin (Table S15), but rather confirmed as parentally inherited SVs which were likely missed due to low coverage (Fig. S5).

### Single nucleotide variants

In addition to SVs, our sequencing depth allowed us to identify SNVs in all five trios form LRS data. We identified, on average, 3.33 million substitutions per genome, of which 23,672 were located in the coding regions (Table S16). Detailed comparison for Trio 5 for all SNVs called from SRS and LRS showed substantial overlap. Over 95% of SNVs identified in SRS were also identified in LRS. In the coding regions, the overlap was more pronounced, reaching 97% (Table S17). Furthermore, we looked at the transition/transversion ratio (Ti/Tv) of called SNVs, which is often used as a metric to detect biases. SNVs called in both LRS and SRS data had a T/Tv ratio of 2.1, which is in line with expectations regarding WGS data [44], suggesting that most variants are genuine biological events (Table S17). In contrast, SNVs uniquely detected by SRS or LRS had Ti/Tv ratios of 1.17 and 0.99, respectively, which may indicate a higher degree of false positive calls in these sets. Evaluation of MIE rates for SNVs on LRS data showed that for Trio 5, with 40× coverage, the MIE rate was as low as 3%, while for Trios 1–4 it was around 8% (Table S18). The MIE rate in the SRS data of Trio 5 was only 1.5%, almost half of that obtained for the LRS, which demonstrates the overall higher accuracy for SNV calling in SRS.

We overlaid the identified coding SVs with the coding SNVs called in each individual in order to find potential compound heterozygous SV-SNV pairs affecting the same gene and potentially explaining recessive disease inheritance. In total, we found 13 total SV-SNV pairs, but none were likely causal for the patient phenotype (Table S19).

### De novo SNV discovery

Because we could have potentially missed de novo SNVs in poorly covered regions when we performed exome and genome SRS [23, 25], we also identified potential de novo SNVs in all trios based on the LRS data. For this analysis, we used a minimum quality score of 30 as a threshold, as all previously identified de novo point mutations (DNMs) from SRS had scores above this threshold in the LRS data (Tables S1 and S20). We considered missense and loss-of-function mutations as potentially damaging de novo candidates. This resulted in 67 candidate DNMs across all 5 trios (Table S21), in addition to the six de novo variants reported previously [32] (Tables S1 and S20).

### De novo SNV validation

Routine Sanger sequencing validations confirmed 58 of 73 (79%) of those candidate variants. Confirmed variants had significantly higher average quality scores than variants that could not be confirmed in the proband (quality scores 67 versus 40 respectively, *p* = 8.25e−4, Welch two-sample *T*-test). Similar to the LRS SV validations, all Sanger sequencing-confirmed variants, apart from the six already known de novo SNVs, appeared inherited from one of the parents.

### Genome variation in previously uncovered regions

LRS is expected to sequence across genomic regions that are difficult to assess using SRS. Therefore, we identified regions in the complete genome of Trio 5 proband that lacked sequence coverage in only one technology. Genome-wide we found that on average 191.7 Mb of the reference genome remains uncovered by both technologies, including 229.3 Kb of protein-coding sequence and 319.4 Kb telomeric and centromeric sequence (Table S22, Fig. 2). We found an additional 35.2 Mb, including 634.9 Kb of protein-coding sequence corresponding to 105 genes, only covered in LRS data. Vice versa, only 12.5 Mb were uniquely covered by the SRS data, including 20.1 Kb of coding sequence. We compared these results to Ebbert et al.’s [22] study on “dark” gene regions that cannot be adequately be assembled or aligned using standard SRS. Of the 35.2 Mb that is missed by the SRS data in our study, a substantial portion (67%) overlapped with the regions that are identified in Ebbert et al. (Table S22d).

**Fig. 3:**
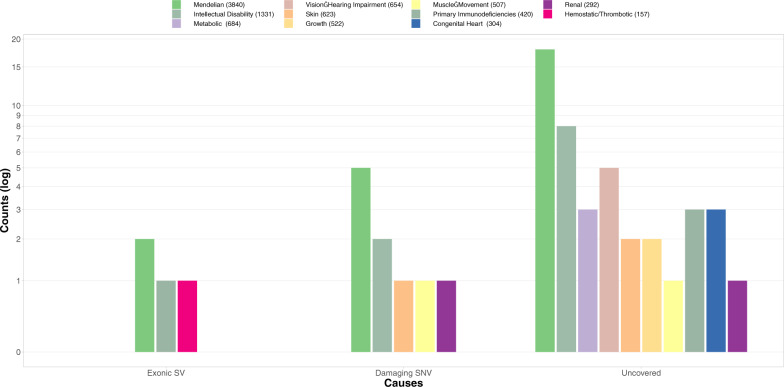
Interpretation of variation identified by LRS but not SRS. From left to right showing three groups: the number of genes of which coding regions are affected by an SV, the number genes affected by a putatively damaging SNV identified only in LRS, the number of genes of which coding regions are covered less than 10% in SRS but that do have coverage in LRS. Individual bars indicate different types of disorders based on diagnostic gene panels as used by Genome Diagnostics Nijmegen (https://www.radboudumc.nl/en/patientenzorg/onderzoeken/exome-sequencing-diagnostics/information-for-referrers/exome-panels), genetic testing laboratory. Numbers in the legend indicate the total number of genes in each of the gene panels.

Importantly, in the 35.2 Mb of LRS-only regions we identified on average 3874 SVs and 32,540 high-quality substitutions in the proband, of which 50 and 672, respectively, were overlapping with genic regions in the proband of Trio 5 (Fig. 3). These 672 genic substitutions included 171 missense and 3 loss-of-function variants (Table S23) and occurred within 43 different genes, including two known genes associated with ID and four other OMIM morbid genes (Table S24).

An additional 378 genes, of which 26 have an established disease-association in OMIM, were only partly covered by SRS (no coverage for more than 10% of the coding sequence; Table S25) but were well-covered in LRS. As expected, we found that these genes had a higher GC-content than genes that were well-covered by SRS (48.8% compared 46.3%, *p*-value < 2.2e−16 Welch two-sample *T*-test).

## Discussion

We performed LRS for five trios with unresolved ID and identified thousands of SVs. We identified an average of 28,292 SVs, of which up to 93% was shown to adhere to mendelian inheritance in five trios. We found considerable overlap with the HG002 published dataset, but slightly less overlap for insertions identified by Audano et al. We expect that this may be because Audano et al. used a different variant calling algorithm (SMRT-SV) whereas variants from HG002 were also called using PBSV. In addition, Audano et al. sequenced mostly individuals of non-European descent.

Although a substantial part of these SVs could not be identified by SRS using three different and commonly used calling algorithms, the significant overlap with existing LRS datasets and Mendelian inheritance concordance indicates that most of these events are likely true events. These SVs were not only present in the most repetitive regions of the genome, but also affected genes and coding regions. We also compared the SV datasets from the three different calling algorithms for SRS to each other, and found that the concordance among these algorithms is disappointingly low (Table S26). This is illustrative of the complexity of SV detection using SRS data, as also observed by others [45] and suggests that LRS technology may be a prerequisite for reliable SV detection. In line with this we estimated that LRS has almost five times higher sensitivity for the detection of SVs than SRS. One striking observation is that the number of detected inversions in our cohort is relatively low, with 28 events in 5 trios compared to other studies that identified 156 inversions per genome [46]. This suggests that sensitivity for these events could be improved, either with improved detection algorithms or alternative sequencing approaches such as Strand-seq [47] or Bionano technology [48].

Notwithstanding the higher raw sequencing error rate of LRS, the relatively high sequence coverage for our samples, allowed us to call SNVs as well. Surprisingly, our SNV calls were of relatively high quality with MIEs as low as 3% for Trio 5, even without the use of Circular Consensus Sequencing technology (CCS) [39]. Most impressive is that all previously identified de novo SNVs through SRS were also identified as potential de novo SNVs in the LRS data, albeit with varying quality scores. SNV calling accuracy would have likely been improved significantly had we been able to use CCS technology [39]. This shows the potential of LRS to comprehensively identify all genome variation in a single experiment.

We hypothesized a de novo SV, previously missed by microarray, exome and genome sequencing may be the cause for the disease in the five individuals with ID sequenced here [23, 32]. However, in this study, we did not identify any de novo SVs that could be confirmed by alternative techniques. There are several possibilities for our lack of confirmed de novo SV events. First of all, the original event in the proband may have been missed due to lack of sequence coverage. Based on an in silico titration and repeated SV calling, we find that although the quality and quantity of the SV yield increases with coverage, beyond 10× the increase in yield diminishes (Table S27).

Secondly, we found that the analyses for the identification of SVs is still being actively developed and results may change considerably depending on the calling algorithm, its version and settings and the reference genome version that is used. Improvements in read alignment and SV calling algorithms for LRS constitute a developing field and future reanalysis of our data may still identify genuine de novo SVs. However, even with such improvements some events may remain too complex to be reliably identified and different technologies may be required.

Thirdly, it is also possible that we have in fact identified de novo SVs but that the methods that were used to validate these events are not reliable enough to confirm such complex forms of genetic variation; for four events, no conclusions could be drawn, showing the need for robust validation methods in these complex genome regions. Lastly, the lack of an identified de novo SV may simply be because de novo mutation rate for SVs is very low. For instance, the current estimate for de novo mutations of large CNVs, is as low as 0.2 events per genome per generation [49]. For other SV types such estimates are less well-established, but it is not unlikely that these are as low as CNVs. Larger cohorts of trio-based LRS are required to fully capture the per generation de novo mutation rates of other structural events.

Alternatively, our initial hypothesis of a previously undetected SV as the cause for ID in these patients, may be incorrect, and the disorder is caused by other types of variants that have so far eluded detection. These could for example be small insertion/deletion events, repeat expansions, or mosaic variation. Moreover, we may have identified the causative variants but have not been able to interpret them correctly.

Although LRS may indeed identify more variation, it is still unknown how much of this genome variation is clinically relevant, and thus how much of an advantage LRS offers over SRS for clinical WGS. Our results show that LRS identifies more SVs across the genome than SRS, some of which affect coding regions, and provides sequence coverage in difficult regions of the genome that harbor protein-coding genes. Using LRS, we are also able to identify SNVs in these regions, and genes within these regions are part of virtual disease gene panels used in routine diagnostic labs for clinical exome interpretation. Whereas we did not identify clinically relevant SNVs in these genes, it is not unreasonable to speculate that there are patients out there that will benefit from variant calling in these regions only accessible by LRS. With the technological advances of CCS marketed as HiFi reads [39] shortly, further enhancing robustness of SNV calling in LRS data, one may expect that genome-wide LRS may allow comprehensive analysis of all variant types per individual genome for clinical and genetic heterogenous disorders such as ID in the future.

### Web resources

UCSC genome browser: https://genome.ucsc.edu/ Integrative Genomics Viewer (IGV): https://software.broadinstitute.org/software/igv/.

## Supplementary information

Supplemental Materials

Table S6B

Table S21

Table S23D

Table S24

Table S25

Clinical Descriptions

## Data Availability

Data for all samples are accessible at EGA under accession number EGAS00001004319.
