# Supplementary material for: Long-read trio sequencing of individuals with unsolved intellectual disability
Source: Eur J Hum Genet. 2020 Nov 30;29(4):637–48. doi: 10.1038/s41431-020-00770-0 (PMC8115091; doi:10.1038/s41431-020-00770-0)
Supplement: Supplementary file 1 — Supplemental Materials [file 41431_2020_770_MOESM1_ESM.docx]

# Supplemental Data

# Supplemental Figures


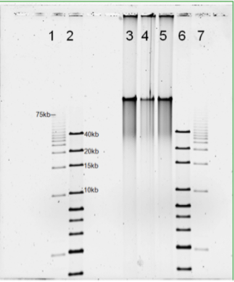


**Figure S1a**. DNA integrity of Trio 5 by Pippin Pulse (Sage Science Inc.). Figure indicates 7 different lanes. (1) DNA size standard 5 Kb ladder (BioRad); (2) 1 kb DNA Extension ladder (Life Tech); (3) Patient DNA sample (T5P); (4) Paternal DNA sample (T5F); Maternal DNA sample (T5M); 1 Kb DNA Extension ladder (Life Tech); (7) DNA size standard 5 Kb ladder (BioRad).


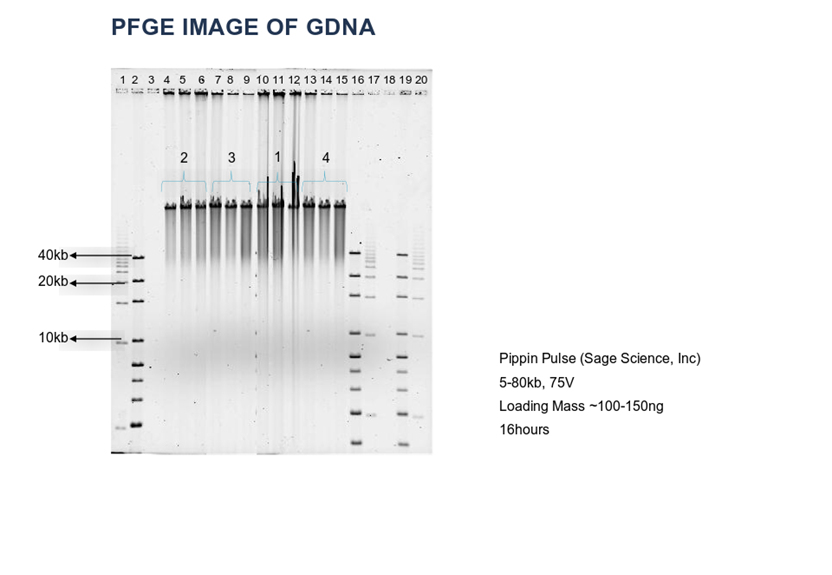


**Figure S1b.** DNA integrity of Trios 1 to 4 (Sage Science Inc.). Lanes are as follows:
Lane 1, 17, 20: 5 Kb ladder (BioRad).
Lane 2, 16, 19: 1 Kb extension ladder (Life Tech)
Lanes 4-15, in order: T2P, T2F, T2M, T3P, T3F, T3M, T1P, T1F, T1M, T4P, T4F, T4M (gDNA)


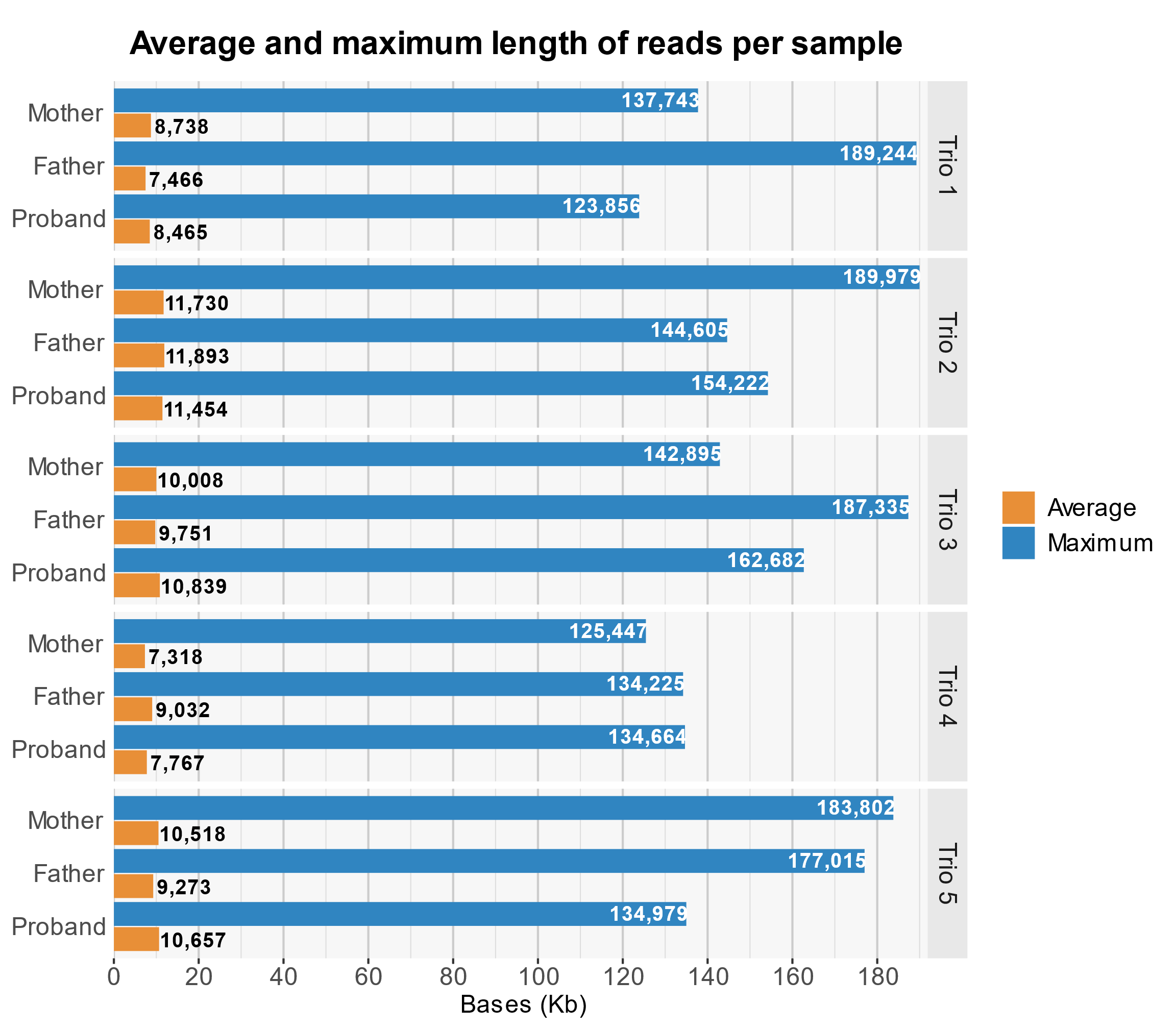


**Figure S2**. Average and maximum read lengths from long-read sequencing shown for the Mother, Father and Proband sample (y-axis) of each trio (panels). The average length is shown in orange, the maximum length in blue.


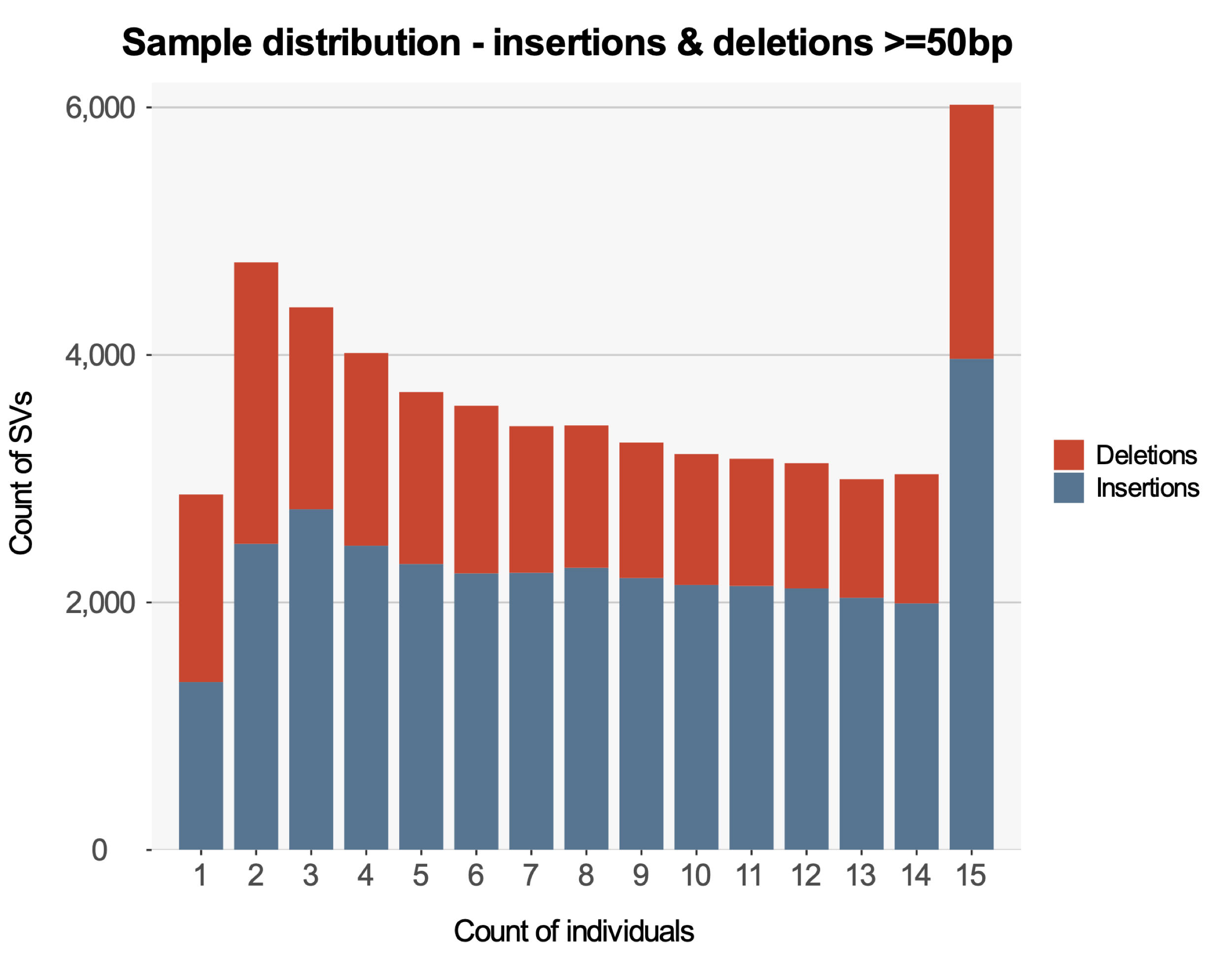


**Figure S3**. Sample distribution of insertions and deletions larger than or equal to 50 bp. The horizontal axis shows the number of individuals sharing an insertion (green) or deletion (red). The vertical axis shows the corresponding count of insertions and deletions.


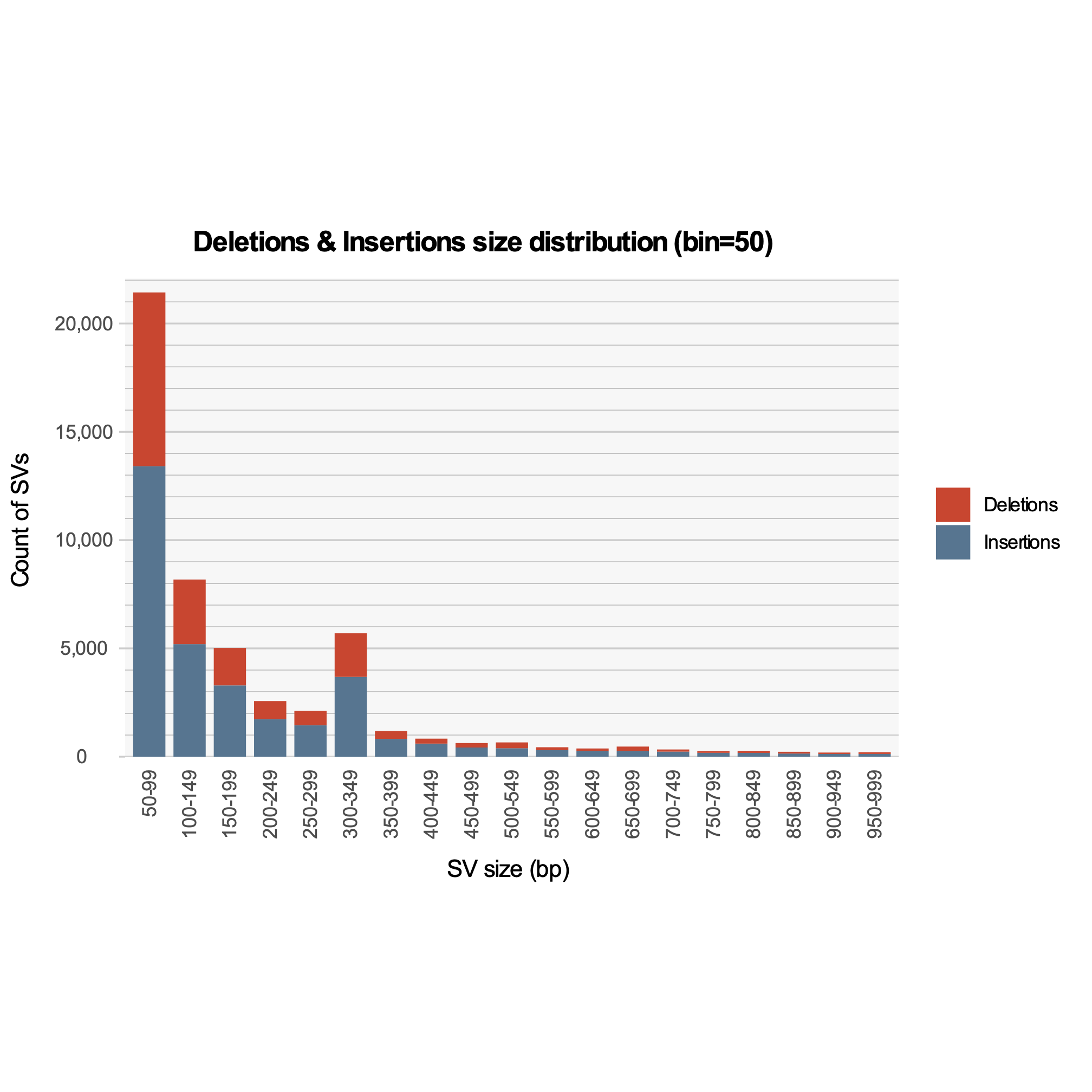


**Figure S4a**. Size distribution of deletions and insertions with size equal to or larger than 50 bp and smaller than 1,000 bp. The count of insertions and deletions (y-axis) is shown for each SV size bin (x-axis). Bin size is 50 bp. Insertions are shown in blue, deletions in red.


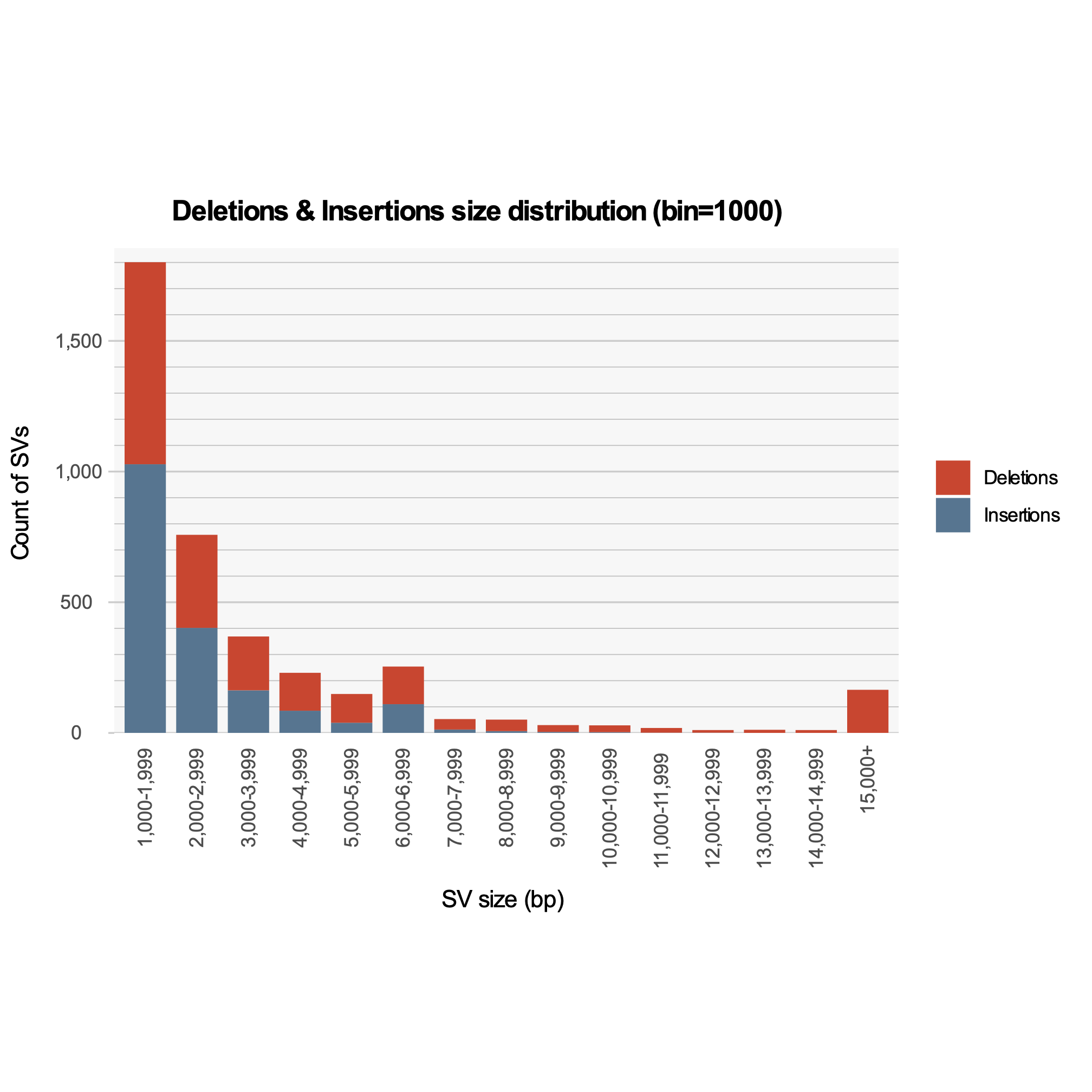


**Figure S4b**. Size distribution of deletions and insertions with size equal to or larger than 1,000 bp. The count of insertions and deletions (y-axis) is shown for each SV size bin (x-axis). Bin size is 1,000 bp until SV sizes smaller than 15,000 bp. Structural variations with size equal to or larger than 15,000 bp are all in the last bin “15,000+”. Insertions are shown in blue, deletions in red.

**Figure S5a**. Overview of long-read alignments spanning an insertion call (SV length: 131 bp, highlighted in red) in sample T2P (top panel). Middle and lower panel correspond to alignments of samples T2M and T2F, respectively. Blue blocks indicate insertions, horizontal black lines indicate deletions. This insertion was only called in the proband of Trio 2, however it was discarded as a *de novo* candidate after this visual inspection. Insertions can be seen all around the region of interest in all three samples.

**Figure S5b**. Overview of long-read alignments spanning an insertion call (SV length: 115 bp, highlighted in red) in sample T3P (top panel). Middle and lower panels correspond to alignments of samples T3M and T3F, respectively. Blue blocks indicate insertions. This insertion was only called in the proband of Trio 3. This structural variant was selected for PCR validation but was not confirmed.

*
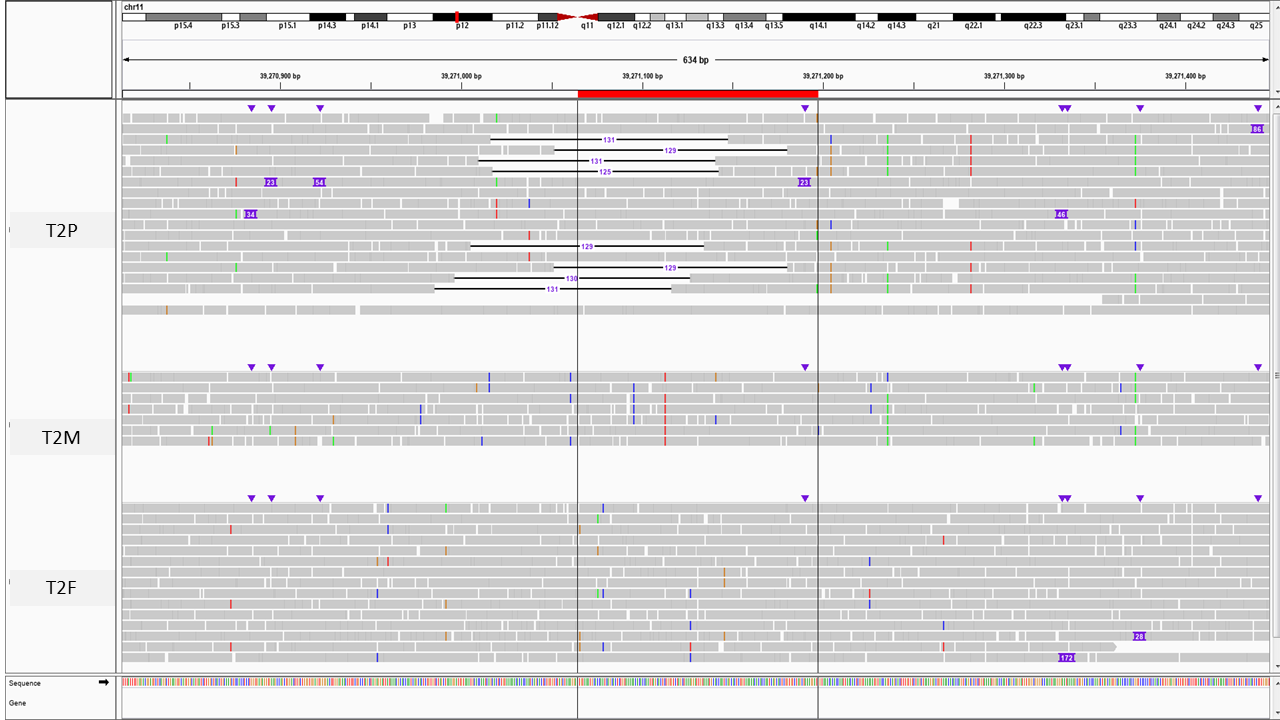
*

**Figure S5c.** Overview of long-read alignments spanning a deletion call (SV length: 133 bp) in sample T2P (top panel). Middle and lower panels correspond to alignments of samples T2M and T2F, respectively. Blue blocks indicate insertions, horizontal black lines indicate deletions. This deletion was only called in the proband of Trio 2 (T2P), but PCR validation showed that it was maternally inherited (T2M).

#
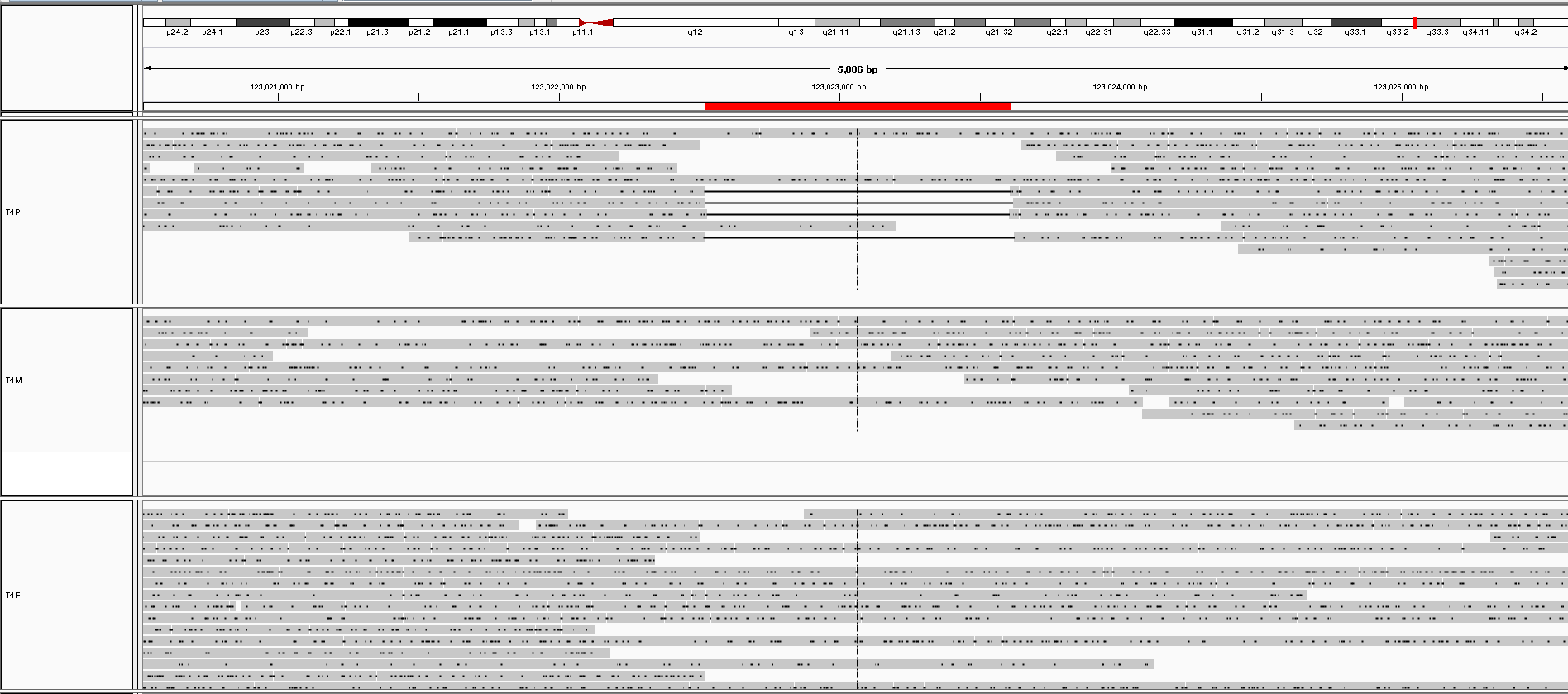


# Figure 5d: Overview of long-read alignments spanning a deletion call (SV length: 1,090 bp) in sample T4P top panel). Middle and bottom panels correspond to alignments of samples T4M and T4F, respectively.

# Supplemental Tables

## Table S1. Overview of sequenced trios and results of previously identified de novo mutations by microarray, WES and WGS.

1. Conversion to previously published data of the same trios

| **Trio ID in current manuscript** | **Trio ID in Gilissen et al. 2014** | **Trio ID de Ligt et al. 2012** |
| --- | --- | --- |
|  |  |  |
| 1 | 14 | 27 |
| 2 | 8 | 19 |
| 3 | 10 | 21 |
| 4 | 47 | 97 |
| 5 | 19 | 35 |

1. Previously published SNVs, indels and CNVs from SRS-based WES and WGS data of these trios

| **Trio ID in current manuscript** | **250 K SNP array (Affymetrix)** | **Gene** | **Genomic annotation (GRCh37)** | **Genomic annotation (GRCh38)** | **cDNA** | | **Protein** |
| --- | --- | --- | --- | --- | --- | --- | --- |
| 1 | Negative | *GPR52* | NC_000001.10:g.174417259T>G | NC_000001.11:g.174448121T>G | NM_005684.4:c.10T>G | p.(Ser4Ala) | |
| 2 | Negative | *CNOT1* | NC_000016.9:g.58564225C>A | NC_000016.10:g.58530321C >A | NM_016284.3:c.6204G>T | p.(Leu2068=) | |
| 3 | Negative | *LIPI* | NC_000021.8:g.15535845C>T | NC_000021.9:g.14163524C >T | NM_198996.2:c.965-1G>A | p.(?) | |
|  |  | *MFAP3* | NC_000005.9:g.153429439A>G | NC_000005.10:g.A154049879>G | NM_005927.4:c.157A>G | p.(Ser53Gly) | |
| 4 | Negative | *ABCC8* | NC_000011.9:g.17483205G>A | NC_000011.10: g.17461658G >A | NM_000352.3:c.747C>T | p.(Ala249=) | |
| 5 | Negative | *TBKBP1* | NC_000017.10:g.45786784A>C | NC_000017.11:g.47709418A >C | NM_014726.2:c.1685A>C | p.(His562Pro) | |

**Table S2.** Per sample long-read sequencing and mapping metrics. Columns indicate (from left to right) the sample name (M: mother, F: father, P: proband), number of SMRT cells used, total number sequenced bases, percentage of bases that mapped to the reference genome and mean mapping quality of those alignment, median read length, N50 of read lengths, mean fold coverage of reference genome and per-base error rate.

| **Sample** | **SMRT Cells** | **Bases sequenced (x10^9^) ^a^** | **% Bases mapped ^a^** | **Mean mapping quality ^b^** | **Mean read length ^a^** | **Median read length ^a^** | **N50 read length^a^** | **Mean Coverage (x) ^a^** | **Error rate ^b^** |
| --- | --- | --- | --- | --- | --- | --- | --- | --- | --- |
| T1F | 10 | 39.98 | 94.59 | 51.70 | 7,466 | 4,494 | 14,553 | 12.60 | 0.1689 |
| T1M | 12 | 46.15 | 96.62 | 52.07 | 8,738 | 5,671 | 16,248 | 14.86 | 0.1651 |
| T1P | 13 | 48.34 | 97.27 | 51.91 | 8,465 | 5,158 | 16,478 | 15.67 | 0.1625 |
| T2F | 10 | 43.26 | 98.50 | 52.23 | 11,893 | 8,654 | 20,620 | 14.20 | 0.1489 |
| T2M | 13 | 52.39 | 98.72 | 52.25 | 11,730 | 8,557 | 20,185 | 17.24 | 0.1501 |
| T2P | 11 | 52.80 | 98.07 | 52.15 | 11,454 | 8,308 | 20,241 | 17.26 | 0.1505 |
| T3F | 12 | 56.34 | 97.48 | 51.99 | 9,751 | 6,452 | 18,205 | 18.30 | 0.1567 |
| T3M | 11 | 55.32 | 97.63 | 52.09 | 10,008 | 6,648 | 18,619 | 18.00 | 0.1550 |
| T3P | 12 | 45.79 | 98.40 | 52.06 | 10,839 | 7,326 | 19,793 | 15.02 | 0.1557 |
| T4F | 12 | 51.82 | 96.83 | 51.86 | 9,032 | 5,847 | 16,935 | 16.72 | 0.1595 |
| T4M | 15 | 50.99 | 94.9 | 51.96 | 7,318 | 4,423 | 14,181 | 16.13 | 0.1681 |
| T4P | 14 | 50.21 | 96.39 | 52.12 | 7,767 | 4,829 | 14,741 | 16.13 | 0.1635 |
| T5F | 30 | 116.49 | 96.93 | 51.88 | 9,273 | 7,136 | 15,208 | 37.64 | 0.1638 |
| T5M | 28 | 123.23 | 97.52 | 52.19 | 10,518 | 7,852 | 18,056 | 40.06 | 0.1599 |
| T5P | 31 | 128.14 | 97.42 | 52.19 | 10,657 | 8,004 | 18,160 | 41.61 | 0.1603 |

^a^**:** Calculated using SAMtools

**^b^**: Calculated using Qualimap

**Table S3.** Percentage of genome covered at a specific minimum fold coverage with long-read sequencing. Columns (from left to right) indicate the sample name and percentage of the genome that is covered at different specific fold-coverage.

1. Genome-wide

| **Sample** | **1x** | **5x** | **10x** | **15x** | **20x** | **30x** |
| --- | --- | --- | --- | --- | --- | --- |
| T1P | 87.18 | 85.2 | 70.81 | 33.17 | 6.89 | 0.60 |
| T1F | 87.08 | 81.98 | 49.24 | 11.78 | 1.62 | 0.37 |
| T1M | 86.47 | 85.03 | 67.11 | 26.21 | 4.77 | 0.49 |
| T2P | 87.14 | 85.75 | 77.14 | 47.97 | 14.79 | 0.75 |
| T2F | 87.12 | 84.73 | 66.66 | 26.82 | 4.77 | 0.48 |
| T2M | 86.50 | 85.74 | 79.22 | 48.83 | 14.7 | 0.73 |
| T3P | 87.15 | 85.08 | 69.89 | 31.65 | 6.31 | 0.54 |
| T3F | 87.21 | 85.94 | 78.55 | 52.57 | 18.79 | 0.97 |
| T3M | 86.48 | 85.81 | 80.23 | 51.44 | 16.41 | 0.79 |
| T4P | 86.51 | 85.41 | 72.6 | 34.92 | 8.10 | 0.60 |
| T4F | 87.15 | 85.47 | 74.27 | 41.19 | 11.01 | 0.68 |
| T4M | 86.50 | 85.29 | 71.20 | 32.29 | 7.00 | 0.56 |
| T5P | 87.32 | 86.96 | 86.52 | 85.16 | 82.29 | 67.53 |
| T5F | 87.32 | 86.95 | 86.32 | 84.26 | 80.74 | 54.94 |
| T5M | 86.65 | 86.27 | 85.97 | 85.59 | 84.44 | 64.64 |

1. Excluding decoy sequences, centromeres, assembly gaps and chromosomes Y and M

| **Sample** | **1x** | **5x** | **10x** | **15x** | **20x** | **30x** |
| --- | --- | --- | --- | --- | --- | --- |
| T1P | 99.90 | 98.26 | 82.21 | 38.26 | 7.50 | 0.31 |
| T1F | 99.85 | 94.77 | 57.04 | 13.22 | 1.43 | 0.15 |
| T1M | 99.89 | 98.75 | 78.05 | 30.14 | 5.06 | 0.23 |
| T2P | 99.89 | 98.90 | 89.62 | 55.69 | 16.79 | 0.46 |
| T2F | 99.89 | 97.81 | 77.46 | 30.87 | 5.06 | 0.22 |
| T2M | 99.90 | 99.53 | 92.25 | 56.70 | 16.68 | 0.45 |
| T3P | 99.90 | 98.17 | 81.16 | 36.51 | 6.84 | 0.27 |
| T3F | 99.91 | 99.02 | 91.15 | 61.01 | 21.44 | 0.69 |
| T3M | 99.91 | 99.59 | 93.43 | 59.78 | 18.69 | 0.51 |
| T4P | 99.91 | 99.16 | 84.49 | 40.37 | 8.96 | 0.33 |
| T4F | 99.89 | 98.62 | 86.30 | 47.73 | 12.36 | 0.39 |
| T4M | 99.90 | 99.06 | 82.89 | 37.32 | 7.69 | 0.32 |
| T5P | 99.94 | 99.84 | 99.61 | 98.37 | 95.46 | 78.63 |
| T5F | 99.94 | 99.83 | 99.41 | 97.43 | 93.76 | 63.80 |
| T5M | 99.94 | 99.83 | 99.71 | 99.47 | 98.30 | 75.24 |

**Table S4a.** Number of structural variants identified per sample using long-read sequencing. Columns indicate (from left to right) the number of insertions, deletions, inversions (INV) and the total for all SV types (ALL).

| **Sample** | **Insertions** | **Deletions** | **Inversions** | **Total** |
| --- | --- | --- | --- | --- |
| T1F | 17,053 | 9,409 | 13 | 26,475 |
| T1M | 17,656 | 9,747 | 18 | 27,421 |
| T1P | 19,284 | 9,728 | 18 | 29,030 |
| T2F | 17,779 | 9,615 | 18 | 27,412 |
| T2M | 18,595 | 10,036 | 18 | 28,649 |
| T2P | 18,808 | 9,940 | 18 | 28,766 |
| T3F | 18,869 | 10,085 | 17 | 28,971 |
| T3M | 18,841 | 10,106 | 15 | 28,962 |
| T3P | 18,181 | 9,914 | 16 | 28,111 |
| T4F | 18,673 | 10,058 | 15 | 28,746 |
| T4M | 18,308 | 9,996 | 18 | 28,322 |
| T4P | 18,590 | 10,033 | 17 | 28,640 |
| T5F | 22,057 | 11,205 | 15 | 33,277 |
| T5M | 22,277 | 10,845 | 16 | 33,138 |
| T5P | 22,224 | 10,816 | 16 | 33,056 |

**Table S4b**: Number of SVs identified across all samples per SV type.

| **SV Type** | **SVs** |
| --- | --- |
| Deletions | 20,307 |
| Insertions | 34,690 |
| Inversions | 28 |
| Total | 55,025 |

**Table S5.** Total sequence affected by identified deletions, insertions and inversions.

1. Per sample statistics per SV type.

| **Sample** | **Length of deletions (bp)** | **Length of insertions (bp)** | **Length of inversions (bp)** |
| --- | --- | --- | --- |
| T1P | 6,321,673 | 6,419,269 | 34,517 |
| T1F | 5,579,491 | 5,734,459 | 26,999 |
| T1M | 6,928,885 | 6,036,450 | 33,369 |
| T2P | 6,279,901 | 6,652,360 | 42,045 |
| T2F | 6,269,045 | 6,287,343 | 39,307 |
| T2M | 6,317,910 | 6,489,171 | 44,839 |
| T3P | 6,374,362 | 6,281,624 | 33,534 |
| T3F | 6,912,171 | 6,379,491 | 36,219 |
| T3M | 6,359,273 | 6,492,193 | 33,283 |
| T4P | 6,715,108 | 6,076,872 | 38,143 |
| T4F | 6,198,669 | 6,309,719 | 32,351 |
| T4M | 6,670,428 | 5,976,964 | 35,983 |
| T5P | 6,760,602 | 7,288,536 | 36,254 |
| T5F | 7,138,215 | 7,062,017 | 34,972 |
| T5M | 6,681,198 | 7,233,589 | 34,792 |
| **Average** | **6,500,462** | **6,448,004** | **35,774** |

1. Total unique sequence length affected by SVs across all samples per SV type.

| **SV Type** | **Total length (bp)** |
| --- | --- |
| Deletions | 15,823,531 |
| Insertions | 11,449,711 |
| Inversions | 65,960 |
| **Total** | **27,339,202** |

**Table S6a**. Number of SVs affecting coding regions and known genes. Columns indicate (from left to right) trio name, number of SVs that overlapped with exonic regions, number of exonic SVs in known ID genes, number of exonic SVs in OMIM genes (including the known ID genes).

| Trio | Exonic SVs | Known ID Genes | OMIM Genes |
| --- | --- | --- | --- |
| T1 | 160 | 0 | 10 |
| T2 | 176 | 5 | 20 |
| T3 | 178 | 4 | 23 |
| T4 | 159 | 1 | 12 |
| T5 | 190 | 6 | 19 |
| Total | **530** | **12** | **81** |

**Table S6b:** List of SVs overlapping OMIM genes. Columns indicate (from left to right) chromosome, start and end positions of the SV (GRCh38p13), length of the SV, the affected gene and its transmission mechanism.

External file: Table_S6b.xlsx

**Table S7.** Comparison of SVs detected using LRS across all trios with the HG002 dataset. Columns indicate (from left to right) the type of SV (SV type), the number of SVs shared between the datasets (Shared), the number of SVs only found in this study (RUMC only), the number of SVs only found in the HG002 dataset (HG002 only). The percentages are calculated based on the number of SVs within each study.

| **SV Type** | **This Study total** | **HG002 total** | **Shared** | **This study only** | **% This study only** | **HG002 only** | **% HG002 only** |
| --- | --- | --- | --- | --- | --- | --- | --- |
| Insertions | 34,690 | 9,521 | 7,043 | 27,647 | 79.70% | 2,478 | 26.03% |
| Deletions | 20,307 | 7,650 | 5,920 | 14,387 | 70.85% | 1,730 | 22.61% |
| Inversions | 28 | 9 | 9 | 19 | 67.86% | 0 | 0% |
| **Total** |  |  | **12,972** | **42,053** | **76.43%** | **4,208** | **24.49%** |

**Table S8**. Comparison between this study’s and Audano *et al.*’s SV calls. Columns indicate (from left to right) the SV type, the number of SVs shared between datasets, the number of SVs that are unique to this study, the percentage of SVs that are unique to this study, the number of SVs that are unique to Audano et *al.*’s study, and the percentage of SVs that are unique to Audano et *al.*’s dataset.

| **SV Type** | **Shared** | **This study only** | **% This study only** | **Audano *et al* only** | **% Audano *et al* only** |
| --- | --- | --- | --- | --- | --- |
| Insertions | 5,982 | 15,963 | 72.74% | 49,778 | 89.27% |
| Deletions | 9,358 | 4,526 | 32.59% | 30,069 | 76.26% |
| Inversions | 11 | 17 | 60.71% | 215 | 95.13% |

**Table S9**. Per sample short-read sequencing and mapping metrics for Trio 5. Columns indicate (from left to right) the sample name, total number of bases sequenced, percentage of bases mapped to the reference genome, mean mapping quality of those bases, mean fold coverage and per-base error rate.

| **Sample** | **Bases sequenced (x10^9^) ^a^** | **Bases mapped ^a^ (%)** | **Mean**  **mapping quality ^b^** | **Mean**  **Coverage (x) ^a^** | **Error rate ^b^** |
| --- | --- | --- | --- | --- | --- |
| T5F | 89.40 | 98.71 | 45.43 | 29.42 | 0.0058 |
| T5M | 85.64 | 98.86 | 45.31 | 28.22 | 0.0054 |
| T5P | 89.83 | 98.71 | 45.48 | 29.56 | 0.0057 |

**^a^:** Calculated using SAMtools

**^b^**: Calculated using Qualimap

**Table S10**. Percentage of genome covered at specific fold coverage with short-read sequencing for Trio 5. Columns indicate (from left to right) the sample name and percentage of genome covered at specific fold coverage

1. Genome-wide

| Sample | 1x | 5x | 10x | 15x | 20x | 30x |
| --- | --- | --- | --- | --- | --- | --- |
| T5P | 94.9 | 94.52 | 93.68 | 90.85 | 85.56 | 45.01 |
| T5F | 94.94 | 94.59 | 93.74 | 90.83 | 85.32 | 43.35 |
| T5M | 94.21 | 93.8 | 93.36 | 92.36 | 86.81 | 36.51 |

1. Excluding decoy sequences, centromeres, assembly gaps and chromosomes Y and M

| **Sample** | **1x** | **5x** | **10x** | **15x** | **20x** | **30x** |
| --- | --- | --- | --- | --- | --- | --- |
| T5P | 99.93 | 99.84 | 99.26 | 96.72 | 91.51 | 47.91 |
| T5F | 99.94 | 99.85 | 99.24 | 96.62 | 91.16 | 46.01 |
| T5M | 99.93 | 99.85 | 99.67 | 98.84 | 93.04 | 38.71 |

**Table S11**. Comparison of SV calls between long-read sequencing (LRS) and short-read sequencing (SRS). Columns indicate (from left to right) type of the SV, number of SVs in SRS set, number of SVs in the SRS set that matched to LRS set, percentage of SVs in SRS set that matched to LRS set, number of the SVs in the LRS set, number of SVs in LRS that matched to SRS set, percentage of SVs in LRS that matched to SRS set.

1. Comparison of SV calls between LRS and SRS for Manta SV calling.

|  | **SRS Input** | **LRS Input** | **Match** | **SRS Match %** | **LRS Match %** |
| --- | --- | --- | --- | --- | --- |
| Deletions | 7,445 | 14,703 | 5,560 | 74.68 | 37.82 |
| Insertions | 2,970 | 28,363 | 2,476 | 83.37 | 8.73 |
| Inversions | 747 | 21 | 6 | 0.8 | 28.57 |

1. Comparison of SV calls between LRS and SRS for LUMPY SV calling.

|  | **SRS Input** | **LRS Input** | **Match** | **SRS Match %** | **LRS Match %** |
| --- | --- | --- | --- | --- | --- |
| Deletions | 4,762 | 14,703 | 3,721 | 78.14 | 25.31 |
| Insertions | NA | NA | NA | NA | NA |
| Inversions | 89 | 21 | 6 | 6.74 | 28.57 |

1. Comparison of SV calls between LRS and SRS for DELLY SV calling.

|  | **SRS Input** | **LRS Input** | **Match** | **SRS Match %** | **LRS Match %** |
| --- | --- | --- | --- | --- | --- |
| Deletions | 9,535 | 14,703 | 4,913 | 51.53 | 33.41 |
| Insertions | 311 | 28,363 | 283 | 91.00 | 1.00 |
| Inversions | 672 | 21 | 6 | 0.90 | 28.57 |

**Table S12a**. Per trio Mendelian inheritance errors for deletions, insertions and inversions detected with LRS sequencing. Columns indicate (from left to right) the trio (Trio), the structural variation type (SV Type), the number of structural variants (SVs), the number (MIE) and percentage (MIE %) of Mendelian inheritance errors, and the number of SVs for which Mendelian inheritance could not be determined because of missing genotypes in one or more members of a trio (Undet.). The latter were ignored for the calculation of the percentage of Mendelian inheritance errors.

| Trio | SV Type | SVs | MIE | MIE (%) | Concordance (%) | Undet. |
| --- | --- | --- | --- | --- | --- | --- |
| T1 | Deletions | 13,497 | 874 | 6.51 | 93.49 | 78 |
|  | Insertions | 26,639 | 3,535 | 13.32 | 86.68 | 106 |
|  | Inversions | 20 | 0 | 0 | 100.00 | 0 |
|  | Total | 40,156 | 4,409 | 10.98 | 89.02 |  |
| T2 | Deletions | 13,584 | 720 | 5.33 | 94.67 | 85 |
|  | Insertions | 26,099 | 2,466 | 9.48 | 90.52 | 78 |
|  | Inversions | 24 | 0 | 0 | 100.00 | 0 |
|  | Total | 39,707 | 3,186 | 8.02 | 91.98 |  |
| T3 | Deletions | 13,885 | 688 | 4.98 | 95.02 | 67 |
|  | Insertions | 26,133 | 1,993 | 7.66 | 92.34 | 99 |
|  | Inversions | 19 | 1 | 5.26 | 94.74 | 0 |
|  | Total | 40,037 | 2,682 | 6.70 | 93.30 |  |
| T4 | Deletions | 13,941 | 683 | 4.94 | 95.06 | 108 |
|  | Insertions | 26,167 | 2,080 | 8 | 92.00 | 171 |
|  | Inversions | 20 | 2 | 10 | 90.00 | 0 |
|  | Total | 40,128 | 2,765 | 6.89 | 93.11 |  |
| T5 | Deletions | 14,703 | 646 | 4.41 | 95.59 | 65 |
|  | Insertions | 28,363 | 1,484 | 5.24 | 94.76 | 47 |
|  | Inversions | 21 | 1 | 4.76 | 95.24 | 0 |
|  | Total | 43,087 | 2,131 | 4.95 | 95.05 |  |
| All | Deletions | 69,610 | 3,611 | 5.18 | 94.82 | 403 |
|  | Insertions | 133,401 | 11,558 | 8.66 | 91.34 | 501 |
|  | Inversions | 104 | 4 | 3.84 | 96.16 |  |
|  | Total | 203,115 | 15,173 | 7.47 | 92.53 |  |

**Table S12b**. Mendelian inheritance errors for deletions, insertions and inversions detected in Trio 5 with SRS sequencing with 3 different SV calling tools. Columns indicate (from left to right) the SV calling tool (Tool), the structural variation type (SV Type), the number of structural variants (SVs), the number (MIE) and percentage (MIE %) of Mendelian inheritance errors, and the number of SVs for which Mendelian inheritance could not be determined because of missing genotypes in one or more members of a trio (Skipped). The latter were ignored for the calculation of the percentage of Mendelian inheritance errors.

| **Tool** | **SV Type** | **SVs** | **MIE** | **MIE (%)** | **Skipped** |
| --- | --- | --- | --- | --- | --- |
| **Manta** | Deletions | 7,445 | 511 | 6.86 | 0 |
|  | Insertions | 4,772 | 305 | 6.39 | 0 |
|  | Inversions | 749 | 34 | 4.54 | 0 |
| **LUMPY** | Deletions | 4,736 | 198 | 4.19 | 6 |
|  | Insertions | 802 | 84 | 10.58 | 8 |
|  | Inversions | 80 | 2 | 2.5 | 0 |
| **DELLY** | Deletions | 16,541 | 629 | 3.81 | 28 |
|  | Insertions | 5,249 | 178 | 3.4 | 10 |
|  | Inversions | 674 | 34 | 5.05 | 1 |

**Table S13**. Number of deletions and insertions detected with LRS that are uniquely found in one of the probands and the corresponding Mendelian errors. Columns indicate (from left to right) the type of structural variants (SV Type), the number of structural variants that are unique among the probands (Unique SVs), the number of Mendelian inheritance errors in structural variants that are unique among the probands (MIE), and the percentage of Mendelian inheritance errors in structural variants that are unique among the probands (MIE %).

1. Total in all trios.

| **SV Type** | **Unique SVs** | **MIE** | **MIE (%)** |
| --- | --- | --- | --- |
| Deletions | 4,738 | 439 | 9.27 |
| Insertions | 6,505 | 1,564 | 24.04 |

1. per trio

| **Trio** | **SV Type** | **SVs** | **MIE** | **MIE (%)** |
| --- | --- | --- | --- | --- |
| T1 | Deletions | 856 | 95 | 11.10 |
|  | Insertions | 1,175 | 353 | 30.04 |
| T2 | Deletions | 872 | 77 | 8.83 |
|  | Insertions | 1,397 | 391 | 27.99 |
| T3 | Deletions | 916 | 83 | 9.06 |
|  | Insertions | 1,129 | 314 | 27.81 |
| T4 | Deletions | 985 | 89 | 9.04 |
|  | Insertions | 1,208 | 245 | 20.28 |
| T5 | Deletions | 1,109 | 95 | 8.57 |
|  | Insertions | 1,596 | 261 | 16.35 |

**Table S14.** Candidate *de novo* SVs per trio using strict calling (See Methods). Columns indicate (from left to right) trio in which the de novo SVs were called, number of deletions, insertions, inversions, and the total of *de novo* SV calls.

| **Trio** | **Deletions** | **Insertions** | **Inversions** | **Total** |
| --- | --- | --- | --- | --- |
| T1 | 1 | 13 | 0 | 14 |
| T2 | 4 | 13 | 0 | 17 |
| T3 | 0 | 11 | 0 | 11 |
| T4 | 3 | 4 | 0 | 7 |
| T5 | 0 | 2 | 0 | 2 |
| **Average** | **1.6** | **8.6** | **0** | **10.2** |

**Table S15**. Identified candidate *de novo* SVs and their validation results by PCR. Columns indicate from (left to right): identifier of the trio in which the event was called, chromosome, start and end coordinates (GRCh38.p13), type of SV (deletion, insertion or inversion), length of the event, result of the validation, closest gene to the SV event.

| **Trio** | **Chromosome** | **Start** | **End** | **SV Type** | **Length (bp)** | **Validation** | **Gene** |
| --- | --- | --- | --- | --- | --- | --- | --- |
| T1 | chr17 (NC_000017.11) | 5,057,563 | 5,061,125 | Deletion | 3,562 | Inherited both parents | *AC012146.2* |
| T1 | chr19  (NC_000019.10) | 6,916,521 | 6,916,521 | Insertion | 2,939 | Could not determine | *ADGRE1* |
| T1 | chr7  (NC_000007.14) | 132,282,661 | 132,282,661 | Insertion | 90 | Variant not confirmed | *PLXNA4* |
| T2 | chr11  (NC_000011.10) | 39,271,064 | 39,271,197 | Deletion | 133 | Inherited maternal | *-* |
| T3 | chr6  (NC_000006.12) | 113,596,551 | 113,596,551 | Insertion | 115 | Variant not confirmed | *-* |
| T3 | chr7  (NC_000007.14) | 1,171,594 | 1,171,594 | Insertion | 95 | Could not determine | *-* |
| T4 | chr9  (NC_000009.12) | 123,022,520 | 123,023,610 | Deletion | 1,090 | Inherited paternal | *RABGAP1* |
| T4 | chr9  (NC_000009.12) | 136,598,505 | 136,598,799 | Deletion | 294 | Inherited maternal | - |

**Table S16**. Number of detected single nucleotide variants from LRS and SRS platforms. Columns indicate (from left to right) sample name, number of SNVs detected in the whole genome, percentage of MIEs in the whole genome (for probands), number of SNVs detected in the exome, percentage of MIEs in the exome (for probands).

1. In long-read sequencing

| **Sample** | **SNVs Genome** | **Genome MIE (%)** | **SNVs Exome** | **Exome MIE (%)** |
| --- | --- | --- | --- | --- |
| T1P | 3,142,916 | 14.28 | 22,906 | 12.09 |
| T1F | 2,663,675 |  | 22,186 |  |
| T1M | 3,007,973 |  | 22,900 |  |
| T2P | 3,383,890 | 8.59 | 23,422 | 9.00 |
| T2F | 3,127,690 |  | 22,740 |  |
| T2M | 3,353,748 |  | 23,777 |  |
| T3P | 3,147,660 | 6.97 | 22,825 | 6.78 |
| T3F | 3,382,461 |  | 23,435 |  |
| T3M | 3,352,767 |  | 23,341 |  |
| T4P | 3,166,126 | 9.52 | 23,326 | 8.39 |
| T4F | 3,261,812 |  | 23,582 |  |
| T4M | 3,101,729 |  | 23,263 |  |
| T5P | 3,956,435 | 3.16 | 26,088 | 2.35 |
| T5F | 3,905,927 |  | 25,578 |  |
| T5M | 3,932,800 |  | 25,818 |  |
| **Average** | **3,325,841** |  | **23,672** |  |

1. In short-read sequencing

| **Sample** | **SNVs Genome** | **Genome MIE (%)** | **SNVs Exome** | **Exome MIE (%)** |
| --- | --- | --- | --- | --- |
| T5P | 3,589,003 | 1.50 | 24,819 | 1.29 |
| T5F | 3,585,991 |  | 24,726 |  |
| T5M | 3,563,637 |  | 24,646 |  |
| **Average** | **3,579,544** |  | **24,730** |  |

**Table S17**. Comparison between SNV calls of LRS and SRS platforms on Trio 5. Rows indicate (from top to bottom) number of SNVs uniquely detected by LRS, percentage of the SNVs that are uniquely detected by LRS, Ti/Tv ratio of SNVs that are uniquely detected by LRS. Further rows show the same information for SNVs that are uniquely detected by SNV and SNVs that are common to both platforms, respectively.

| **Sample** | **T5P** | **T5F** | **T5M** |
| --- | --- | --- | --- |
| LRS Only | 511,099 | 481,489 | 514,581 |
| LRS Only (%) | 12.92 | 12.33 | 13.08 |
| LRS Only, Genic | 3,923 | 3,635 | 4,011 |
| LRS Only, Genic (%) | 15.04 | 14.21 | 15.54 |
| LRS Only Ti/Tv | 0.99 | 1 | 1.02 |
| SRS Only | 143,597 | 161,492 | 145,336 |
| SRS Only (%) | 4.00 | 4.50 | 4.08 |
| SRS Only, Genic | 649 | 713 | 656 |
| SRS Only, Genic (%) | 2.61 | 2.88 | 2.66 |
| SRS Only Ti/Tv | 1.17 | 1.25 | 1.26 |
| Both | 3,445,336 | 3,424,438 | 3,418,219 |
| Both Ti/Tv | 2.13 | 2.13 | 2.13 |

**Table S18.** MIE rates of Longshot called SNVs that are unique among probands. Columns indicate (from left to right) trio name, number of SNVs that are unique among probands, number of MIEs in SNVs that are unique among probands, percentage of MIEs in SNVs that are unique among probands.

| Sample | Proband unique | Proband MIE | Proband MIE (%) |
| --- | --- | --- | --- |
| T1 | 1,146 | 170 | 14.83 |
| T2 | 1,239 | 194 | 15.66 |
| T3 | 1,094 | 127 | 11.61 |
| T4 | 1,227 | 103 | 8.39 |
| T5 | 1,198 | 61 | 5.09 |
| Total | **5,904** | **655** | **11.09** |

**Table S19**. Identified bi-allelic SV and SNV combinations that form a compound heterozygote. Columns indicate (from left to right) the sample, the name of the affected gene, the position and the type of the SV, and the position of the damaging SNV.

| Sample | Gene name | SV (GRCh38.p13) | Damaging SNV (GRCh38.p13) | SNV Effect |
| --- | --- | --- | --- | --- |
| T1P | *ENTPD2* | NC_000009.12:g.137050283_137050284ins | NC_000009.12:g.137053898G>T | Stop-gain |
| T1P | *PRH1* | NC_000012.12:g.10882513_10882574del | NC_000012.12:g.11061546G>A | Stop-gain |
| T1P | *PRB4* | NC_000012.12:g.11308619_11308620ins | NC_000012.12:g.11308868C>T | Stop-gain |
| T1P | *GOLGA6L6* | NC_000015.10:g.20534338_20534505del | NC_000015.10:g.20535014G>T | Stop-gain |
| T1P | *GOLGA6L22* | NC_000015.10:g.23327869_23327870del | NC_000015.10:g.23129688G>T | Stop-gain |
| T1P | *GOLGA6L22* | NC_000015.10:g.23334090_23334091ins | NC_000015.10:g.23129688G>T | Stop-gain |
| T1P | *ZNF77* | NC_000019.10:g.2939220_2939290del | NC_000019.10:g.2936537C>T | Stop-gain |
| T2P | *PRH1* | NC_000012.12:g.10882513_10882574del | NC_000012.12:g.11061546G>A | Stop-gain |
| T4P | *PRH1* | NC_000012.12:g.10882513_10882574del | NC_000012.12:g.11061546G>A | Stop-gain |
| T4P | *TXNDC2* | NC_000018.10:g.9887391_9887435del | NC_000018.10:g.9887032G>T | Stop-gain |
| T4P | *FAM71E2* | NC_000019.10:g.55358511_55358534del | NC_000019.10:g.55359721G>T | Stop-gain |
| T5P | *PRH1* | NC_000012.12:g.10882513_10882574del | NC_000012.12:g.11061546G>A | Stop-gain |
| T5P | *ZNF77* | NC_000019.10:g.2939220_2939290del | NC_000019.10:g.2936537C>T | Stop-gain |

**Table S20**. Known *de novo* coding SNVs from previous studies and their quality score in Longshot SNV calling from LRS. Columns indicate (from left to right) the gene in the loci, position of the SNV (GRCh38.p13), substitution and effect of the SNV, sample name and variant call quality score from Longshot.

| Sample | Gene | Position | Variant | Effect | Quality Score |
| --- | --- | --- | --- | --- | --- |
| T1P | *GPR52* | NC_000001.11:g.174,448,121 | T>G | Missense | 78.41 |
| T2P | *CNOT1* | NC_000016.10:g.58,530,321 | C>A | Synonymous | 93.41 |
| T3P | *MFAP3* | NC_000005.10:g.154,049,879 | A>G | Missense | 93.57 |
| T3P | *LIPI* | NC_000021.9:g.14,163,524 | C>T | Splice site | 68.38 |
| T4P | *ABCC8* | NC_000011.10:g.17,461,658 | G>A | Synonymous | 30.74 |
| T5P | *TBKBP1* | NC_000017.10:g.47,709,418 | A>C | Missense | 89.99 |

**Table S21**. Validation results of potential *de novo* SNVs identified in LRS. Columns indicate (from left to right) Variant effect, gene and exon information, chromosome and the position of the variant (GRCh38.p13), reference and allele nucleotides, genotype, quality score and validation results.

**External file:** Table_S21.xlsx

**Table S22**. Regions with no coverage in sample Trio 5 proband and comparison between short-read sequencing and long-read sequencing. Columns (from left to right) show the regions with no coverage using short-read sequencing (SRS), regions with no coverage using long-read sequencing (LRS), and regions with no coverage in both short-read and long-read sequencing (Both).

1. Zero coverage regions across the whole genome.

| Zero Coverage | SRS | LRS | Both |
| --- | --- | --- | --- |
| Number of gaps | 293,002 | 146,996 | 258,646 |
| Total size of gaps (bp) | 35,159,625 | 12,520,500 | 191,679,200 |
| Average size of gaps (bp) | 119 | 85 | 741 |
| Largest gap size (bp) | 499,472 | 4,368 | 30,000,001 |
| Smallest gap size (bp) | 1 | 1 | 1 |
| Telomeres and Centromeres | 52,762 | 288 | 319,384 |

1. Zero coverage regions overlapping within genes.

| Zero Coverage | SRS | LRS | Both |
| --- | --- | --- | --- |
| Number of gaps | 47,146 | 7,871 | 25,759 |
| Total size of gaps (bp) | 7,197,247 | 358,818 | 4,020,023 |
| Average size of gaps (bp) | 152 | 45 | 156 |
| Largest gap size (bp) | 221,296 | 2,694 | 283,290 |
| Smallest gap size (bp) | 1 | 1 | 1 |

1. Zero coverage regions overlapping with exons.

| Zero Coverage | SRS | LRS | Both |
| --- | --- | --- | --- |
| Number of gaps | 5,088 | 422 | 2,448 |
| Total size of gaps (bp) | 634,929 | 20,165 | 229,301 |
| Average size of gaps (bp) | 125 | 48 | 94 |
| Largest gap size (bp) | 4,407 | 1,079 | 8,694 |
| Smallest gap size (bp) | 1 | 1 | 1 |

1. Zero coverage regions overlapping with Ebbert *et al* (2019)

| Zero Coverage | Genomic | Genic | Exonic |
| --- | --- | --- | --- |
| Number of gaps | 229,968 (79%) | 28,239 (59%) | 1,617 (32%) |
| Total size of gaps (bp) | 23,702,114 (67%) | 4,375,469 (61%) | 106,025 (17%) |
| Average size of gaps (bp) | 103 | 155 | 66 |
| Largest gap size (bp) | 34,752 | 22,548 | 4,407 |
| Smallest gap size (bp) | 1 | 1 | 1 |

**Table S23.** Summary of LRS-detected SVs and SNVs in regions not covered by SRS in Trio 5. The variants are further broken down into those in genic and coding regions.

1. Structural variation identified in regions that are not covered coverage in SRS.

| Sample | SVs in SRS gaps | Genic | Exonic |
| --- | --- | --- | --- |
| T5P | 3,893 | 1,660 | 50 |
| T5F | 3,810 | 1,636 | 54 |
| T5M | 3,920 | 1,655 | 54 |
| Average | **3,874** | **1,650** | **53** |

1. High-quality SNVs in regions that are not covered by SRS in Trio 5.

| Sample | SNVs in SRS gaps | Genic | Exonic |
| --- | --- | --- | --- |
| T5P | 34,356 | 11,105 | 672 |
| T5F | 28,574 | 8,978 | 527 |
| T5M | 34,689 | 11,482 | 606 |
| Total | **97,619** | **31,565** | **1,805** |

1. Breakdown of 672 genic SNVs that are detected in trio 5 in regions not covered with SRS. “Unknown” denotes variants of unknown consequence due to lack of ORF annotations.

| **Type** | **Number** |
| --- | --- |
| Missense | 171 |
| Synonymous | 290 |
| Stop gain | 1 |
| Splicing | 2 |
| UTR | 85 |
| ncRNA | 114 |
| Unknown | 9 |

1. List of Exonic SNV only detected by long-read sequencing

**External file:** Table_S23d.xlsx

**Table S24**. Genes that are affected by damaging SNVs in the regions uncovered by SRS. Columns indicate the HGNC gene name and pLI score obtained from GnomAD. OMIM morbid genes are shown in bold.

**External file:** Table_S24.xlsx

**Table S25**. Genes with significant coverage gaps in SRS. Columns indicate (from left to right): gene name(HGNC), total size of the regions (in base pairs) that are uniquely missed by SRS, total size of the regions (in base pairs) that uniquely missed by LRS, total size of the regions (in base pairs) that are missed by both SRS and LRS, size of the exonic regions (in base pairs) that are uniquely missed by SRS, size of the exonic regions (in base pairs) that are uniquely missed by LRS, size of the exonic regions (in base pairs) that are both missed by SRS and LRS and gene length in base pairs. Known ID genes are shown in bold.

**External file:** Table_S25.xlsx**Table S26**. Number of SVs per type identified by exclusive combinations of methods. Columns indicate (from left to right) the method (P: PBSV, D: DELLY, L: LUMPY, M: Manta), the number of deletions, the number of insertions and the number of inversions. A dash indicates non-applicable values when a specific method does not call a specific SV type.

| **Callers** | **Deletions** | **Insertions** | **Inversions** |
| --- | --- | --- | --- |
| P | 8,152 | 25,868 | 15 |
| D | 3,905 | 23 | 265 |
| L | 414 | - | 23 |
| M | 1,435 | 489 | 346 |
| PD | 453 | 19 | 0 |
| PL | 71 | - | 0 |
| PM | 1,458 | 2,212 | 0 |
| DL | 350 | - | 18 |
| MD | 173 | 5 | 353 |
| ML | 83 | - | 12 |
| PLD | 467 | - | 0 |
| PMD | 919 | 264 | 0 |
| PML | 109 | - | 0 |
| MDL | 194 | - | 30 |
| PMDL | 3074 | - | 6 |
| **Total** | **21,257** | **28,880** | **1,068** |

**Table S27**.

1. Precision and recall rate and F1 measure for long-read sequencing SV calling at different fold coverages. Columns indicate (from left to right) fold coverage, total number of SV calls, number SVs that overlap with full coverage call set, precision, recall and F1 score. *We note that these numbers do not correlate with the numbers mentioned for the full cohort since PBSV performs multi-sample calling and gains additional sensitivity when calling larger cohorts (i.e. the full set of 15 samples compared to this single trio).*

| **Coverage (x)** |  |  | **SV Count** | **TP**  **Count** | **Precision** | **Recall** | **F1 Score** |
| --- | --- | --- | --- | --- | --- | --- | --- |
| 5x |  |  | 12,357 | 9,810 | 0.8008 | 0.4699 | 0.5923 |
| 10x |  |  | 19,031 | 16,338 | 0.8597 | 0.7754 | 0.8154 |
| 15x |  |  | 20,495 | 17,505 | 0.8545 | 0.8299 | 0.8421 |
| 20x |  |  | 20,784 | 17,925 | 0.8611 | 0.8479 | 0.8544 |
| 30x |  |  | 21,060 | 18,355 | 0.8699 | 0.8678 | 0.8688 |

1. **Precision and recall rate for SNV calling at different fold coverages. Columns indicate (from left to right)** fold coverage, total number of SNV calls, number of SNVs that overlap with full coverage call set, precision and recall scores.

| Coverage (x) | SNV Count | TP Count | Precision | Recall |
| --- | --- | --- | --- | --- |
| 5x | 1,759,615 | 1,525,202 | 0.8668 | 0.3638 |
| 10x | 3,245,825 | 2,927,112 | 0.9018 | 0.6982 |
| 15x | 3,795,295 | 3,535,649 | 0.9316 | 0.8434 |
| 20x | 3,992,319 | 3,795,936 | 0.9508 | 0.9055 |
| 30x | 4,124,097 | 4,005,418 | 0.9712 | 0.9554 |
