# Supplementary material for: Long-read trio sequencing of individuals with unsolved intellectual disability
Source: Eur J Hum Genet. 2020 Nov 30;29(4):637–48. doi: 10.1038/s41431-020-00770-0 (PMC8115091; doi:10.1038/s41431-020-00770-0)
Supplement: Supplementary file 7 — Clinical Descriptions [file 41431_2020_770_MOESM7_ESM.docx]

**Supplementary Clinical descriptions (based on de Ligt et al. 2012 and Gilissen et al. 2014).**

***Trio 1* -** This boy was born after an uncomplicated pregnancy and delivery. He had a post axial polydactyly of his left hand. From the start he showed passive behavior, feeding problems and recurrent airway infections. At one year and three months he could sit with help and received tube feeding. He had a length of 67.5 cm (<-2.5 SD) and an OFC of 45.5 cm (-1.8 SD). He had a coarse face, bitemporal narrowing, clear blue eyes, epicanthic folds, low-set ears, a short nose and full lips. In addition, he had a sacral dimple and a full post axial polydactyly of his left hand. 250k SNP array analysis and Prader-Willi methylation studies were normal. Analysis of the protein coding sequenced by WES and WGS did reveal a de novo variant in *GPR52*, a gene currently not associated to a disease-phenotype or considered as candidate for the disorder observed in this patient (de Ligt et al. 2012; Gilissen et al. 2014).

***Trio 2* -** This boy was born after a pregnancy complicated by oligohydramnion and maternal thyroid disease. After birth an atrioventricular septal defect and cryptorchidism were noted. Severe developmental delay was present from the start. MRI of the brain at 4.5 months of age was normal. He had recurrent airway infections and feeding problems. During early infancy he had had seizures, however at the age of 3 years and 4 months, these were not present anymore. He had a height of 93 cm (-2 SD), weight of 14.1 kg (0.5 SD) and OFC of 44 cm (<-2.5 SD). He had prominent eyes, a hypoplastic midface and a small penis. 250k SNP array analysis, a metabolic screen and *MECP2*

analyses were normal. Analysis of the protein coding sequenced by WES and WGS revealed a de novo synonymous variant in *CNOT1* (de Ligt et al. 2012; Gilissen et al. 2014). Whereas this gene is recently described to cause a NDD (Vissers et al. AJHG 2020), the synonymous variant in patient 2 did not show any signs of pathogenicity upon functional evaluation, and is, as such, considered to result from the overall per-generation de novo mutation rate and not the explanation of the patients phenotype.

***Trio 3* -** This boy was born after an uncomplicated pregnancy and delivery. At three months of age he developed seizures and West syndrome was diagnosed. He could role at 4 months. At 2 years and 5 months he was not able to sit without support. He had a height of 84 cm (-2.5 SD), weight of 11.5 kg (-1 SD) and OFC of 46 cm (-2.5 SD). He did not show major dysmorphisms, he had mild upslanting palpebral fissures (similar to his father), a small mouth and plagiocephaly. In addition, he was diagnosed with non-symptomatic thrombocytopenia (± 30 à 50 x 109 /l). MRI of the brain showed mild white matter anomalies, cerebellar atrophy and mildly enlarged ventricles. 250k SNP array analysis, *CDKL5, MECP2, ARX, FMR1* testing, and a metabolic screen were normal. Analysis of the protein coding sequenced by WES and WGS revealed two de novo variants, being a canonical splice site variant in *LIPI* and missense variant in *MFAP3* (de Ligt et al. 2012; Gilissen et al. 2014). To date, neither of these genes is associated to NDD, and the observed variants are currently not considered to be the explanation of disease in this patient.

***Trio 4* -** This female was born after an uncomplicated pregnancy and delivery. During her first year of life she had two Apparent Life Threatening Events (ALTE). Since the age of 18 months her delay in development became obvious. She had clumsy motor skills and speech development was severely delayed. At adult age she was able to speak in short, simple sentences. At physical examination at the age of 20 years she had a normal weight and height, but small head circumference of 51.7 cm (<-2.5 SD). She had no evident dysmorphism. SNP array analysis and a metabolic screen were normal. Analysis of the protein coding sequenced by WES and WGS revealed a de novo synonymous change in *ABCC8* (de Ligt et al. 2012; Gilissen et al. 2014). This variant gene is not considered to explain the patients phenotype.

***Trio 5* -** This boy was born after an uncomplicated pregnancy and delivery. A the age of eight months inactive behavior was noted. He could walk without support at fourteen months of age. Since two years of age he had epilepsy. MRI of the brain was normal. At the age of four years and four months he could speak with about 100 single words, without formation of multiple word sentences. He had four to five seizures a day. He had a height of 106.5cm (0 SD) and an OFC of 51cm (0 SD). He had mild down slanting palpebral fissures with long eye lashes, mild prominent ears, short philtrum and full lips. 250k SNP array analysis and *FMR1* testing were normal. Analysis of the protein coding sequenced by WES and WGS revealed a de novo missense variant in *TBKBP1*, which is not expected to explain the patients phenotype (de Ligt et al. 2012; Gilissen et al. 2014).
